# Revealing the crucial roles of suppressive immune microenvironment in cardiac myxoma progression

**DOI:** 10.1038/s41392-024-01912-2

**Published:** 2024-08-02

**Authors:** Zedong Jiang, Qianlong Kang, Hong Qian, Zhijie Xu, Huan Tong, Jiaqing Yang, Li Li, Renwei Li, Guangqi Li, Fei Chen, Nan Lin, Yunuo Zhao, Huashan Shi, Juan Huang, Xuelei Ma

**Affiliations:** 1 Department of Biotherapy, State Key Laboratory of Biotherapy, West China Hospital, Sichuan University, Chengdu, Sichuan China; 2 Frontiers Science Center for Disease-related Molecular Network, West China Hospital, Sichuan University, Chengdu, Sichuan China; 3 Key Laboratory of Bio-Resource and Eco-Environment of Ministry of Education, College of Life Sciences, Sichuan University, Chengdu, Sichuan China; 4 Department of Cardiovascular Surgery, West China Hospital, Sichuan University, Chengdu, Sichuan China; 5 Department of Pathology, Xiangya Hospital, Central South University, Changsha, Hunan China; 6 Institute of Clinical Pathology, West China Hospital, Sichuan University, Chengdu, Sichuan China; 7 Department of Hematology, Sichuan Academy of Medical Sciences and Sichuan Provincial People’s Hospital, University of Electronic Science and Technology of China, Chengdu, China

**Keywords:** Cardiology, Oncogenesis

## Abstract

Cardiac myxoma is a commonly encountered tumor within the heart that has the potential to be life-threatening. However, the cellular composition of this condition is still not well understood. To fill this gap, we analyzed 75,641 cells from cardiac myxoma tissues based on single-cell sequencing. We defined a population of myxoma cells, which exhibited a resemblance to fibroblasts, yet they were distinguished by an increased expression of phosphodiesterases and genes associated with cell proliferation, differentiation, and adhesion. The clinical relevance of the cell populations indicated a higher proportion of myxoma cells and M2-like macrophage infiltration, along with their enhanced spatial interaction, were found to significantly contribute to the occurrence of embolism. The immune cells surrounding the myxoma exhibit inhibitory characteristics, with impaired function of T cells characterized by the expression of *GZMK* and *TOX*, along with a substantial infiltration of tumor-promoting macrophages expressed growth factors such as *PDGFC*. Furthermore, in vitro co-culture experiments showed that macrophages promoted the growth of myxoma cells significantly. In summary, this study presents a comprehensive single-cell atlas of cardiac myxoma, highlighting the heterogeneity of myxoma cells and their collaborative impact on immune cells. These findings shed light on the complex pathobiology of cardiac myxoma and present potential targets for intervention.

## Introduction

Cardiac myxoma, the most prevalent type of cardiac tumor, is generally considered benign, due to the slow proliferation rate and lack of metastasis.^1,2^ Despite its benign nature, the strategic location of cardiac myxomas within the heart and their inherent pathological features often result in significant clinical manifestations. Patients frequently experience embolic complications, where tumor fragments or thrombi dislodge and travel through the bloodstream, leading to severe and sometimes life-threatening conditions such as stroke, systemic embolization, or infarction of vital organs.^3^ Currently, surgical resection remains the only available therapeutic option for patients with cardiac myxoma.^4^ This procedure involves the complete removal of the tumor from the heart, typically through open-chest surgery, which, although effective, carries inherent risks. The invasiveness of the surgery means that some patients, particularly those with comorbidities or advanced age, may not be suitable candidates due to the increased risk of perioperative morbidity and mortality.^5^ Furthermore, potential postoperative complications, such as infection, bleeding, and arrhythmias, can significantly impact patient recovery and quality of life. Additionally, cardiac myxoma tends to recur, especially in those individuals with a familial history of disease.^6,7^ Therefore, it is imperative to acquire a comprehensive understanding of the underlying disease mechanisms, identify potential drug targets, and develop alternative pharmacological interventions to enhance the efficacy of current treatment strategies for cardiac myxoma.

Cardiac myxomas are mostly round or oval, with significant individual variation in size, ranging from a few millimeters to several centimeters in diameter. They typically have a short stalk attached to the fossa ovalis region of the atrial septum. Some myxomas lack a stalk and are diffusely adhered to the heart wall. Pathologically, cardiac myxoma cells usually appear as single entities or clustered together, forming mucinous cord-like and tubular structures.^8^ Previous research suggests that cardiac myxomas may originate from multipotent cardiac stem cells located at the fossa ovalis and endocardium. Early studies have indicated that stromal cells from cardiac myxomas can express the von Willebrand factor endothelial marker, diffuse cytoplasmic neuropeptides, S100 protein, and neuron-specific enolase, providing evidence that myxomas may arise from multipotent mesenchymal cells with neuroendocrine and endothelial differentiation potential.^9^ Further research has isolated c-kit + /CD45-/CD31- cells from cardiac myxomas, demonstrating clonal, self-renewing, and sphere-forming stem cell characteristics, thereby confirming the presence of tissue-specific myxoma-initiating stem cells within the tumors.^10^

Several studies have elucidated the molecular characteristics of cardiac myxomas, highlighting features related to cell adhesion, proliferation, and angiogenesis. Genes *MIA* (Melanoma inhibitory activity) and *PLA2G2A* (phospholipase A2 group II) show higher specificity as cardiac myxoma markers and certain gene-expressed protein products such as S-100, human calbindin 2, thrombomodulin, basic fibroblast growth factor, fibroblast growth factor receptor 1, transcription factor 9, and transmembrane receptor protein 1 are overexpressed in cardiac myxomas.^5^ Most of these proteins are associated with tumor proliferation, angiogenesis, and malignant metastasis. Matrix metalloproteinases are overexpressed in papillary myxomas, leading to extracellular matrix degradation and tumor embolization.^11^ Researchers have reported that *MEF2D* (Myocyte enhancer factor 2D), as a transcription factor, is crucial in tumor development, with studies indicating that *MEF2D* is associated with myxoma proliferation, infiltration, and tumor size.^12^ MicroRNAs also play a significant role in the progression of cardiac myxomas.^13^ The downregulation of miR-335 can lead to the de-repression of its target genes, such as *RUNX2*. This activates a reparative mesenchymal stem cell phenotype characterized by enhanced proliferation, migration, and differentiation capabilities, which clinically can result in the formation of myxomas.^10^ And miR-217 is downregulated in the tissues of patients with cardiac myxoma and overexpression of miR-217 inhibited the proliferation of primary myxoma cells and promoted their apoptosis.^14^

However, the cellular composition of cardiac myxomas remains unclear, particularly regarding the immune infiltration environment surrounding the tumors. The identification of drug targets is still in its early stages. There is an urgent need to dissect the molecular and cellular landscape of cardiac myxomas. Single-cell sequencing is currently widely applied in various heart diseases, elucidating crucial cell types associated with the onset of these conditions.^15^ In this study, we employed single-cell sequencing technology to construct a comprehensive cellular atlas of cardiac myxoma and dissected the diverse states exhibited by various cell populations within the myxoma tissue. Our research discovered and defined myxoma cells that demonstrated a distinct gene expression profile from other cells characterized by an increased expression of phosphodiesterases and genes associated with cell proliferation, differentiation, and adhesion. Immune cells surrounding the myxoma displayed suppressive characteristics, potentially contributing to the tumor’s strategy of evading immunity, as evidenced by the impaired effector function of CD8 + T cells expressing *GZMK* and *TOX* highly. Furthermore, our study has revealed that M2-like macrophages expressing *PDGFC* promote the growth of cardiac myxoma cells. The significant impact of myxoma cells and M2-like macrophages on the occurrence of embolism underscores the clinical relevance of those cells in cardiac myxoma patients. Overall, our findings contribute to the advancement of the understanding regarding the complex interactions among different cell populations in cardiac myxoma and provide potential targets for further investigations and therapeutic interventions.

## Results

### scRNA-seq reveals the cellular landscape of cardiac myxomas

To characterize the cellular and functional landscape of cardiac myxomas, we obtained cardiac myxoma tissues from 5 patients with cardiac myxomas (Supplementary Table 1) and performed scRNA-seq using the 10X Genomics 3ʹ Single Cell platform. After filtering low-quality cells and putative doublets, a total of 75,641 cell transcriptomes were retained for subsequent analysis. We integrated the scRNA-seq data from 5 individuals using the Harmony algorithm, and Harmony-corrected principal components were then employed to generate a unified UMAP embedding space (Supplementary Fig. 1a).

**Fig. 1:**
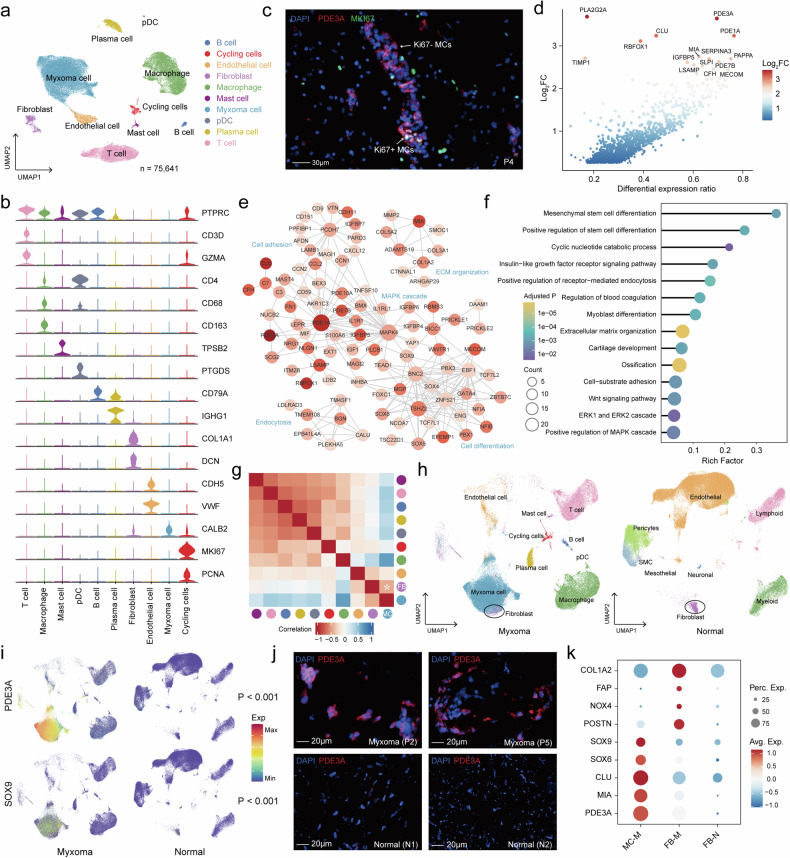
Cell composition of cardiac myxomas. **a** The UMAP plot presents all sequenced cells based on cell type from 5 patients. **b** Violin plots show the expression levels of classic markers across different clusters. **c** The mIHC image displays Ki67+ and Ki67- myxoma cells. Scale bars, 30 μm. **d** The scatter plot shows differential genes between MC and other cell types. Genes with corrected p values less than 0.05 in the differential analysis are displayed in the plot. The x-axis represents the difference in gene expression proportions between the two cell groups, while the y-axis represents the fold difference in gene expression means between the two cell groups. **e** The functional network is formed by marker genes of the MC cluster. Edges in the network represent the semantic similarity of GO annotations between genes. **f** A lollipop chart shows functional enrichment analysis results of marker genes from MC. **g** Heatmap shows the correlation of gene expression between various cell types. **h** Two separate UMAP plots depicting cell types in myxoma and normal heart. The left side represents 5 myxoma samples, annotated in this study. The right side represents 2 normal heart samples, annotated from a published study. **i** Two separate UMAP plots depicting gene expression of *PDE3A* and *SOX9* in myxoma and normal heart. **j** The mIHC image displays PDE3A+ cells in myxoma and normal tissues. Scale bars, 20 μm. **k** The dot plot shows the scaled expression of selected genes for each cluster, colored by the average expression of each gene in each cluster scaled across all clusters. Dot size represents the percentage of cells in each cluster with more than one read of the corresponding gene

The cells were classified into 10 major cell clusters based on graph clustering methods and classical marker annotation (Fig. 1a, b, Supplementary Fig. 1b), including abundant myxoma cells (n = 37,044, 49.0%) identified by the expression of *CALB2* (encoding calretinin, a previously reported myxoma cell marker,^16^) macrophage (n = 17,375, 23.0%) identified by *CD68* and *CD163* expression, T cells (n = 12,012, 15.9%) which expressed the classical T/NK cell marker *CD3D* and granzyme genes, endothelial cells (n = 2675, 3.5%) identified by the expression of *CDH5* and *VWF*, plasma cells (n = 2326, 3.1%) which displayed high expression of *IGHG1*, fibroblasts (n = 1981, 2.6%) marked by *COL1A1* and *DCN*, proliferating cells (n = 943, 1.2%) identified by the expression of cell cycle-related genes (Supplementary Fig. 1c), B cells (n = 626, 0.8%) marked by *CD79A*, mast cells (n = 461, 0.6%) defined by their classical markers *TPSB2*, and a small number of plasmacytoid dendritic cells (pDC, n = 198, 0.3%) positive for *PTGDS* expression. Further analysis revealed that the cells in the proliferative phase mainly contained T/NK cells, macrophages, and myxoma cells (Fig. 1c, Supplementary Fig. 1d, e). Overall, there was minimal proliferation of myxoma cells, and there was heterogeneity among patients (Supplementary Fig. 1f).

We discovered the differentially expressed genes for each cluster (Supplementary Fig. 1g, Data S1), and the results were consistent with the previously identified markers for each cell type. In addition, we identified additional cell markers and transcriptomic features in previously unidentified myxoma cells (Fig. 1d). One of the most notable findings was significant upregulation of cyclic nucleotide phosphodiesterase (PDE) gene families, including *PDE3A*, *PDE1A*, *PDE7B*, and *PDE10A* in myxoma cells (Supplementary Fig. 2a). Functional association network of marker genes revealed that myxoma cells have the capabilities of cell adhesion, extracellular matrix remodeling, insulin-like growth factor regulation, and cell differentiation (Fig. 1e). Co-expression analysis of marker genes revealed gene modules associated with similar functions (Supplementary Fig. 2b, c). Subsequent functional enrichment analysis of myxoma cell markers identified multiple biological processes, such as pathways involved in mesenchymal stem cell differentiation, cyclic nucleotide catabolic process, regulation of receptor-mediated endocytosis, regulation of blood coagulation, extracellular matrix organization, and some signaling pathways including insulin-like growth factor receptor signaling, Wnt signaling, ERK, and MAPK cascade (Fig. 1f, Data S2). We found myxoma cells have a high similarity with fibroblasts (Fig. 1g). Compared with fibroblasts, myxoma cells downregulate the markers of fibroblasts such as *COL1A1* and *DCN* but still retain some abilities of collagen secretion (Supplementary Fig. 2d–f).

### Differences in the microenvironment between myxoma and normal hearts

To compare the cellular composition of myxoma tissue with that of a normal heart, we integrated the scRNA-seq data from healthy adult human hearts obtained from previous studies (6 donors, 85,802 cells) ^17^with the scRNA-seq data from myxoma tissues (5 donors, 75,641 cells). The integrated heart atlas identified 18 major cell subsets, which were annotated based on the original data annotation and classical markers (Supplementary Fig. 3a, b). We found a strong correlation between the two sources of immune cells and a low correlation between stromal cells (Supplementary Fig. 3c). The myxoma tissue displayed a higher abundance of B cells and plasma cells and showed a lower presence of smooth muscle cells, vascular endothelial cells, and pericytes (Fig. 1h, Supplementary Fig. 3d). Myxoma cells derived from myxoma were found to be independent of normal heart tissue and formed a distinct subset in the integrated profile, with high expression of the myxoma cell marker such as *PDE3A* and the transcription factor *SOX9*, which regulate cell differentiation (Fig. 1i). Immunofluorescence experiments confirmed the presence of a significant number of PDE3A+ cells within myxoma tissues, while they were virtually absent in normal cardiac tissues. (Fig. 1j).

We then compared fibroblasts from myxomas with those from normal hearts. (Supplementary Fig. 3e, Data S3). Compared with normal fibroblasts, myxoma fibroblasts expressed myxoma cell markers, but at a lower level of expression (Fig. 1k). Fibroblasts within myxomas exhibit an activated state, characterized by elevated expression of well-known activated fibroblast markers ^18^such as *POSTN*, *NOX4*, *FAP*, and *COL1A2* (Fig. 1k). In addition, myxoma up-regulated genes were associated with the Wnt signaling pathway and mesenchymal cell differentiation (Supplementary Fig. 3f). On the other hand, down-regulated genes were associated with cellular oxidative detoxification, collagen metabolism, and response to oxidative stress (Supplementary Fig. 3g).

### The association between cell types and the severity of cardiac myxomas

Although all cell types were presented in all patients, the proportion of these cell types varied greatly among individuals, possibly reflecting differences in the stage of myxoma progression. Therefore, we embarked on an extensive study to explore the relationship between the cellular composition of cardiac myxoma and its clinical impact. In this regard, we retrospectively gathered data from 49 patients who underwent cardiac myxoma resection at Xiangya Hospital between 2019 and 2022 (Supplementary Table 2), and samples were divided into three groups based on disease severity. Demographic characteristics and echocardiographic findings displayed no significant distinctions among the groups (Table 1). Additionally, we incorporated normal atrial septal from 8 donors as controls. Our investigation aimed to discern whether the cellular composition within myxoma tissues is linked to the severity of cardiac myxoma.

**Table 1:** Clinical characteristics of patients with cardiac myxoma

	Asymptomatic (n = 11)	Mild (n = 31)	Severe (n = 8)	P value
Sex				
Female	8 (72.7%)	23 (74.2%)	6 (75%)	1
Male	3 (27.3%)	8 (25.8%)	2 (25%)	
Age				
<55	4 (36.4%)	17 (54.8%)	4 (50%)	0.615
≥55	7 (63.6%)	14 (45.2%)	4 (50%)	
Hypertension				
No	7 (63.6%)	28 (90.3%)	6 (75%)	0.104
Yes	4 (36.4%)	3 (9.7%)	2 (25%)	
Smoking				
No	9 (81.8%)	26 (83.9%)	6 (75%)	0.871
Yes	2 (18.2%)	5 (16.1%)	2 (25%)	
Size^a^				
<1500 mm^2^	7 (70%)	22 (71%)	8 (100%)	0.268
≥1500 mm^2^	3 (30%)	9 (29%)	0 (0%)	
Location				
Left atrium	10 (90.9%)	26 (83.9%)	8 (100%)	0.884
Right atrium	1 (9.1%)	4 (12.9%)	0 (0%)	
Others	0 (0%)	1 (3.2%)	0 (0%)	
Mobility^a^				
Low	5 (50%)	9 (29%)	1 (12.5%)	0.268
High	5 (50%)	22 (71%)	7 (87.5%)	
Blockage^a^				
No	7 (70%)	17 (54.8%)	5 (62.5%)	0.77
Yes	3 (30%)	14 (45.2%)	3 (37.5%)	

^a^One patient had missing echocardiographic data

**Fig. 2:**
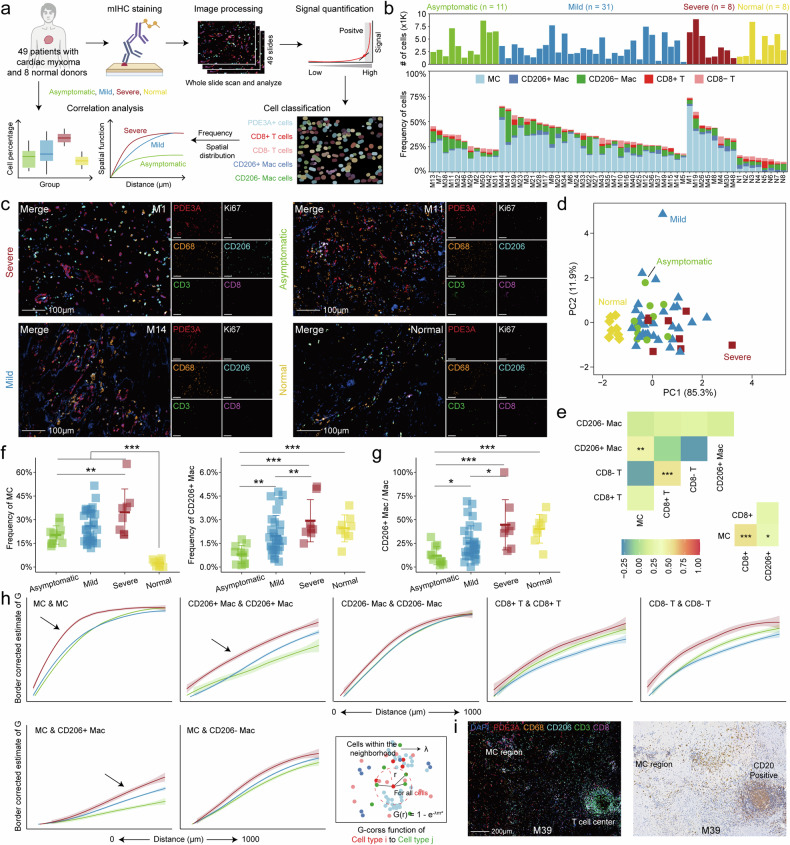
Variability in cell type distributions across the severity of cardiac myxomas. **a** Schematic depicting the analysis of the correlation between cell types and myxoma severity. **b** Barplot depicting the cell numbers (top panel) and distribution (bottom panel) of 5 cell types in 57 samples. MC, myxoma cell; Mac, macrophage. **c** Representative images of antibody staining across severity subgroups. Scale bars, 100 μm. **d** PCA dimension reduction plot based on the proportions of 5 cell types for all samples. The severity of the disease is displayed in different colors and shapes. **e** Heatmap of the correlation between the proportions of each cell type (top panel). Correlation between the proportion of MC and the proportion of CD8 + T/T and CD206 + Mac/Mac (bottom panel). *P < 0.05, **P < 0.01, and ***P < 0.001. **f** Prevalence of MC and CD206+ Mac across asymptomatic (n = 11), mild (n = 31), severe (n = 8) patients, and normal samples (n = 8) as a proportion of total cells. **g** Prevalence of CD206+ Mac/Mac across each subgroup. **h** G-cross curves of cell type *i* to cell type *j* (labeled *i* & *j*) f*i*tted based on different severity subgroups. **i** The mIHC (right panel) and IHC (CD20; right panel) staining demonstrates the T cell center in myxoma. Scale bars, 200 μm

We obtained formalin-fixed paraffin-embedded resection specimens from patients with cardiac myxoma. Utilizing antibodies against PDE3A, Ki67, CD3, CD8, CD68, and CD206, we employed multiplex immunohistochemical staining to investigate the composition of myxoma cells, T cells, and macrophages in each patient (Fig. 2a, b). Proportions of myxoma cells, CD8 + T cells, CD8- T cells, CD206+ macrophages, and CD206- macrophages showed significant variability across patients (Fig. 2c, Supplementary Fig. 4a, Data S4). Based on the cellular ratios, we observed a relative separation trend between severely symptomatic and asymptomatic patients in the PCA dimension reduction plot (Fig. 2d). Correlation analysis highlighted an association between the proportions of myxoma cells and CD206+ macrophages and a correlation between the proportions of myxoma cells and the proportion of CD206+ macrophages in macrophages (Fig. 2e). Interestingly, patients with severe symptoms exhibited significantly higher proportions of myxoma cells and CD206+ macrophages in comparison to asymptomatic patients, while there were no significant differences observed in CD206- macrophage infiltration between the two groups (Fig. 2f, Supplementary Fig. 4b). Furthermore, severely symptomatic patients displayed a notably higher proportion of CD206+ conversion in macrophages (Fig. 2g). On the contrary, PDE3A+ cells were absent in normal tissues, while CD206+ macrophages exhibited a relatively high proportion (Fig. 2f, g). T cell infiltration and proportions of both states showed no statistically significant differences across different symptomatic groups (Supplementary Fig. 4c). Smaller myxomas often displayed a significantly higher proportion of CD8 + T cells (Supplementary Fig. 4d), suggesting their potential cytotoxic function in restricting myxoma size, although that is unrelated to the embolic impact of myxomas.

Subsequently, we investigated the relationship between cellular spatial arrangement and the severity of myxoma. Our findings revealed that interactions among myxoma cells were more compact in the severe group, which is accompanied by a denser distribution of CD206+ macrophages (Fig. 2h, Supplementary Fig. 4e). Conversely, the distances between CD206- macrophages exhibited no significant variation across different groups. Similarly, the proximity between myxoma cells and CD206+ macrophages was closer in the severe group, while distances between myxoma cells and CD206- macrophages showed no intergroup differences (Fig. 2h). Furthermore, we have observed a phenomenon of T cell clustering in certain samples, where myxoma cells were pushed aside, resembling tertiary lymphoid structures ^19^(Fig. 2i), which underscores the pivotal role of T cells in combating myxoma cells.

### Characterizing the functional diversity of myxoma cells

**Fig. 3:**
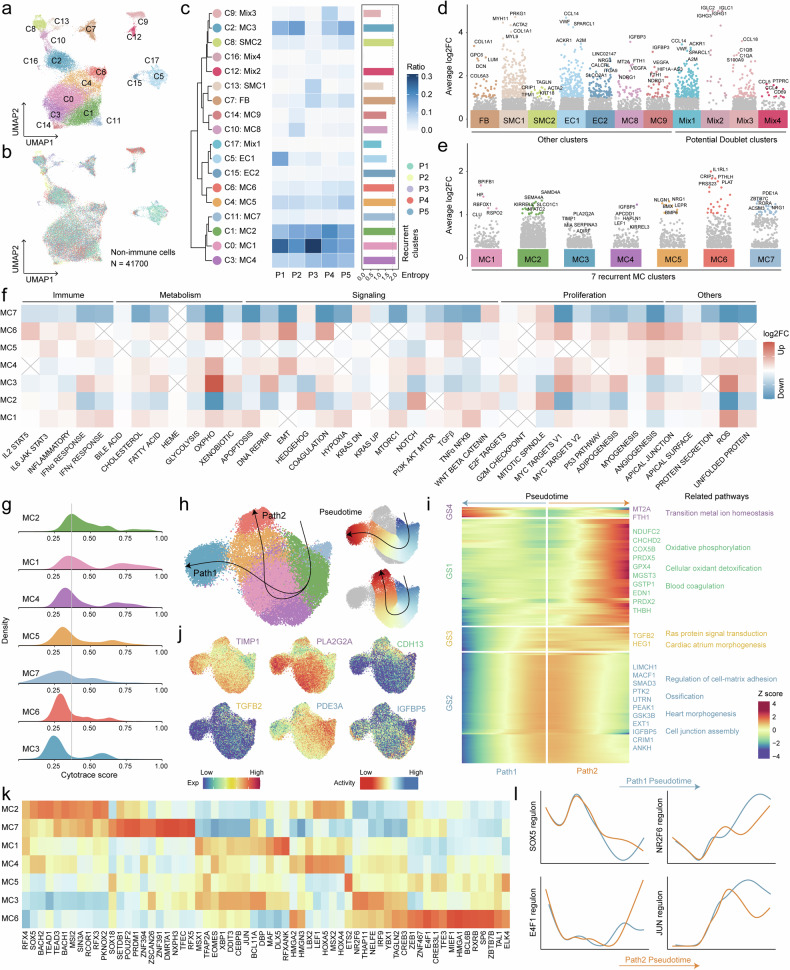
Heterogeneity of nonimmune cells in cardiac myxomas. **a** UMAP view of nonimmune cell clusters from 5 samples. **b** The UMAP plot shows the sample origin of all nonimmune cells. **c** Dendrogram demonstrating the similarity of nonimmune cell cluster centroids. The heatmap shows the proportion of each nonimmune cell subset in different samples. **d**, **e** Marker genes for various nonimmune cell types. The y-axis represents the expression fold change after log_2_ transformation, and genes with log_2_FC > 1 are filled in respective subgroup colors. The top 5 marker genes are annotated in the figure. **f** Heatmap shows log_2_ transformed fold change of hallmark gene set score across 7 recurrent MC clusters. **g** Distribution of CytoTRACE scores for various MC subpopulations. **h** Developmental trajectory of MCs visualized by UMAP. Potential differentiation trajectories are schematically depicted with arrows. The pseudotime of cells is visualized by UMAP (right). **i** Heatmaps **i**llustrate genes linked to developmental path 1 (left) and path 2 (right). These genes were divided into 4 classes based on hierarchy clustering. Representative genes and associated pathways of each gene cluster are labeled. **j** The expression profile of genes related to cell fate is visualized by UMAP. **k** Heatmap shows differential TF activity across MC clusters revealed by SCENIC. **l** Changes in TF activity along pseudotime of two differentiation paths

Since we observed a remarkable divergence of non-immune cells between myxoma and normal heart ecosystems, we embarked on a study to investigate the functional diversity of non-immune cells in myxomas. We re-analyzed the subpopulations of myxoma cells, fibroblasts, and endothelial cells subpopulations by sub-clustering, and a total of 41,700 cells formed 18 clusters (Fig. 3a, Supplementary Fig. 5a). Though most subpopulations were present in all samples, there were some specific to certain samples, and entropy was then used to describe the distribution of each subgroup (Fig. 3b, c, Supplementary Fig. 5b). By calculating the markers of each subpopulation and comparing them to the normal cardiac atlas, we annotated the various subpopulations (Fig. 3d, e, Supplementary Fig. 5c–g, Data S5). Some subsets expressed certain immune cell markers (CD45 and immunoglobulin genes) and were considered potential doublets (Mix1-4). Based on the annotated information of classical markers, we identified two subsets of smooth muscle cell subsets (SMC1 and SMC2) expressing *MYH11* and *TAGLN* that may be involved in vascular formation and one subset of fibroblast. Endothelial cells, which typically express *EPCAM1* and *VWF* were split into two major subpopulations (EC1 and EC2). EC1 highly expresses the venous marker *ACKR1*,^20^ which suggested that EC1 was regarded as vascular endothelial cells and primarily of venous origin. On the other hand, a small portion of EC2 may be endothelial cells derived from arteries (Supplementary Fig. 5g).

In contrast to malignant tumor cells, myxoma cells showed low inter-tumor heterogeneity, with the emergence of seven prevalent MC subsets (Fig. 3e). MC1 was characterized by high expression of tumor proliferation-related genes such as *BPIBF1*, *RSPO2*, and *CLU*, in which *RSPO2* is also associated with stem cell self-renewal.^21^ MC2 exhibits elevated expression of *NFATC2*, which is related to cell cycle regulation.^22^ MC3 is primarily characterized by the expression of functional proteins, including *PLA2G2A*, which is involved in phospholipid metabolism, *TIMP1*, which is related to extracellular matrix remodeling, and *ADIRF*, which regulates adipocyte differentiation and metabolism. MC4 showed high expression of genes such as *IGFBP5*, *APCDD1*, and *HAPLN1*, which play roles in regulating cell proliferation and differentiation, and they are closely linked to tumor progression.^23–25^ The marker genes of MC5 are associated with specific signaling pathways: *NLGN1* and *NRG1* regulate neural cell proliferation, differentiation, and synapse formation, potentially playing a crucial role in heart development;^26,27^ *BMX*, as a tyrosine kinase, participates in cell signaling and regulates cell proliferation and migration;^28^ *LEPR* is involved in leptin signaling; and *BMP6* is involved in cell differentiation, playing roles in tumor progression and immune regulation.^29^ MC6, apart from upregulating genes related to cell proliferation and tumor progressions like *IL1RL1*, *CRIP1*, *PTHLH*, and *PPSS23*, also demonstrates elevated expression of *PLAT*, a gene reported to degrade the extracellular matrix, aiding cancer cells in breaching tissue barriers to promote invasion and metastasis.^30^ MC7 is primarily characterized by the expression of genes such as *PDE1A*, *ZBTB7C*, *RORA*, and *ACSM3*.

Gene set enrichment analysis and hallmark activity analysis revealed the diversity of MC subpopulations (Fig. 3f, Supplementary Fig. 5h). The highly expressed genes in MC1 were associated with complement activation and exhibited elevated levels of apoptosis, tumor necrosis factor signaling, and IFNγ response, suggesting that MC1 may represent a major subgroup involved in immune responses. MC2 is characterized by pathways related to cyclic nucleotide degradation and demonstrates elevated levels of Hedgehog, NOTCH, and WNT signaling, reflecting stem cell maintenance and differentiation characteristics.^31^ MC7 displayed a downregulated KRAS pathway and heightened G2M checkpoint activity, indicating certain growth inhibitory features.^32^ MC3 is associated with the negative regulation of proteolysis and adipocyte differentiation, with strong capabilities in DNA repair and adipogenesis. MC5 is linked to the regulation of chondrogenesis and the response to fibroblast growth factor stimulation, which features high IL6 and TGFβ signaling pathway scores and shows certain immunoevasion traits.^33,34^ The marker genes of MC4 are involved in negative regulation of muscle tissue development, while MC6 is closely related to blood coagulation and may participate in thrombus formation in cardiac myxoma patients; both MC4 and MC6 exhibit strong epithelial-mesenchymal transition (EMT) activity. Additionally, two other sample-specific MC subgroups are associated with cellular responses to low oxygen levels and glycolysis processes (Supplementary Fig. 5h).

### Myxoma cells exhibit two different fate paths

Given the previously observed differentiation capacity of myxoma cells, we aimed to infer the evolutionary trajectory of these cells. Initially, we utilized CytoTRACE ^35^scores to assess the differentiation potential across different subpopulations. The results indicated a diverse range of differentiation potentials among MC subgroups, with a higher differentiation potential in MC2 and MC1 subgroups, while MC3 and MC6 exhibited relatively lower differentiation potential (Fig. 3g).

Then we employed the Slingshot ^36^algorithm to analyze and describe the evolutionary trajectory of mucinous tumor cells. The trajectory inference revealed two distinct cellular evolution paths (Fig. 3h). Combining this information with the differentiation potential of each subgroup, it became evident that MC2 was poised to differentiate into both MC3 and MC6 lineages. Subsequently, we established pseudotime for individual cells along these two paths and utilized the TradeSeq ^37^algorithm to analyze gene expression changes over pseudotime (Data S6). We identified key genes that might drive cellular differentiation along each developmental trajectory. The most relevant top 200 genes were grouped into four distinct gene sets (GS) (Fig. 3i, Supplementary Fig. 6a–e). GS1 displayed increasing expression along differentiation path 2, featuring genes like *NDUFC2*, *COX5B*, *GPX4*, and *CDH13*, which were linked to oxidative phosphorylation, cellular oxidant detoxification, and blood coagulation processes (Fig. 3j, Supplementary Fig. 6a). Meanwhile, GS2 demonstrated diminished expression along both differentiation paths, encompassing genes such as *PDE3A*, *IGFBP5*, and others associated with the regulation of extracellular matrix adhesion, osteogenic differentiation, and cardiac morphogenesis (Fig. 3j, Supplementary Fig. 6b). Conversely, the expression of GS3 decreased along differentiation path 1, including genes like *TGFB2* and *HEG1*, involved in Ras signaling transduction and atrial morphogenesis (Fig. 3j, Supplementary Fig. 6c). The expression of GS4 gradually increased along differentiation path 1, encompassing representative genes such as *TIMP1*, *PLA2G2A*, and several genes associated with transition metal ion homeostasis (Fig. 3j, Supplementary Fig. 6d).

Given the pivotal role of transcription factors (TFs) in cell fate determination, we employed the SCENIC algorithm to unveil the stage-specific TF regulation within each MC subgroup. The results revealed distinct active TFs across subgroups (Fig. 3k). Specifically, *SOX5*, implicated in regulating embryonic development and cell fate determination,^38^ as well as *MSI2*, which governed stem cell self-renewal and differentiation,^39^ displayed prominent activity in the early phases of MC subgroups (Fig. 3k). However, their regulatory activity gradually diminished along pseudotime in both differentiation paths (Fig. 3l, Supplementary Fig. 6f). TFs involved in cell growth and stress response,^40^ such as *JUN*, and those associated with the regulation of lipid metabolism,^41^ such as *NR2F6*, exhibited increased activity in the later stages of both differentiation paths, especially in the late stages of differentiation path 1. In the final stages of differentiation path 2, TFs with elevated regulatory roles included *CREB3L1*, implicated in regulating cellular stress adaptation,^42^ and *TAGLN2*, associated with cell adhesion, migration, and muscle cell function.^43^ These findings underscore the dynamic and intricate regulatory landscape orchestrated by TFs at different stages of mucinous tumor cell differentiation.

### T cells in myxoma tissues exhibit dysfunction

**Fig. 4:**
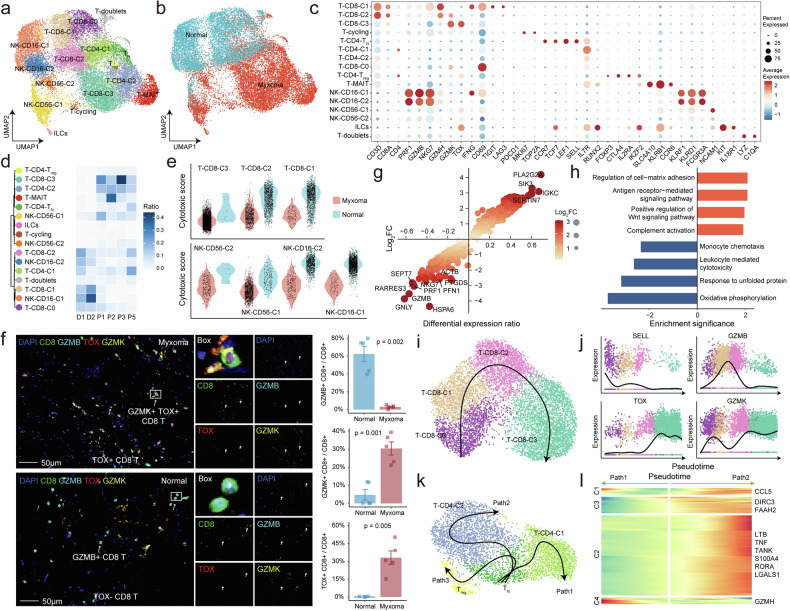
T cell dysfunction in cardiac myxomas. **a** UMAP view of lymphocyte clusters from 6 samples. **b** The UMAP plot shows the group information of all lymphocytes. **c** Expression levels and frequencies of selected markers across lymphocyte clusters. **d** Dendrogram demonstrating the similarity of lymphocyte cluster centroids. The heatmap shows the proportion of each lymphocyte subset in different samples. **e** Violin plots showing cytotoxic scores in CD8 + T and NK cells across myxoma and normal tissues. **f** The mIHC staining demonstrates the CD8 + T cells and *TOX*, *GZMK*, and *GZMB* expression in myxoma and normal tissue. Scale bars, 50 μm. The bar plots show the proportion of T cells expressing *GZMB*, *GZMK*, and *TOX*, n = 5 in each group. The error bar indicates the standard error of the mean. **g** Scatter plot showing differential genes of myxoma and normal tissue–derived lymphocyte cells. Genes with corrected p values less than 0.05 in the differential analysis are displayed in the plot. The x-axis represents the difference in gene expression proportions between the two cell groups, while the y-axis represents the fold difference in gene expression means between the two cell groups. **h** Barplot shows significantly upregulated (orange) and downregulated (blue) gene sets in lymphocytes of myxoma as obtained from the GSEA method. **i** Developmental trajectory of CD8 T cells visualized by UMAP. Potential differentiation trajectory is schematically depicted with arrows. **j** Changes in gene expression along pseudotime of CD8 T cell differentiation trajectory. **k** The developmental trajectory of CD4 T cells is visualized by UMAP, and three differentiation trajectories are schematically depicted with arrows. **l** Heatmaps illustrate genes linked to developmental path 1 (left) and path 2 (right) of CD4 T cell differentiation trajectories. These genes were divided into 4 classes based on hierarchy clustering. Representative genes of each gene cluster are labeled

Prior studies have suggested a crucial role of immune cell infiltration in different tumor niches, and we sought to investigate the adaptive changes in immune cells during the progression of cardiac myxoma. Therefore, we integrated immune cells of cardiac myxoma tissue and published normal adult heart ^17^to further analyze the cell states of all immune cells and compare the differences. Regrouping of lymphocytes identified a total of 16 clusters (Fig. 4a, Supplementary Fig. 7a, b), of which there were apparent differences in the cell state distribution of T and NK cells between normal and myxoma tissues (Fig. 4b).

Among CD8 + T cells, classic effector T cells were present in both myxoma tissues and normal cardiac tissues. CD8 + T cells in normal cardiac tissues exhibited heightened cytotoxicity, characterized by the expression of *IFNG*, *GZMB*, and *GZMH*, as well as a tissue-resident phenotype indicated by *CD69* expression ^44^(Fig. 4c–e, Supplementary Fig. 7c). In contrast, myxoma tissues contain a specific group of CD8 + T cells which is characterized by high expression of *TOX* ^45^and concomitant inflammatory molecule *GZMK*,^46^ which indicates the presence of T cells with impaired function (Fig. 4c–e). The mIHC experiments revealed that compared to normal tissue, myxoma exhibited a higher proportion of CD8 + T cells expressing *TOX* and higher expression levels of *GZMK* while showing lower expression levels of *GZMB* (Fig. 4f), which confirmed the characteristic dysfunction of CD8 + T cells in myxoma. Additionally, we found that CD8 + T cell dysfunction is not significantly correlated with the severity of cardiac myxoma (Supplementary Fig. 7e), suggesting that CD8 + T cell dysfunction is a common phenomenon in myxoma. Moreover, myxoma tissues contain a distinct subset of mucosal-associated invariant T (MAIT) cells, which may be associated with antibacterial host defense.^47,48^ NK cells encompassed two subsets including CD16 + NK and CD56 + NK, where CD16 + NK cells predominate in normal cardiac tissues, and CD56 + NK cells are the principal subset in myxoma tissues. Furthermore, NK cells in myxomas exhibit reduced cytotoxicity (Fig. 4e, Supplementary Fig. 7d).

Lymphocytes within myxoma tissues exhibit elevated expression of certain genes associated with extracellular matrix adhesion, while their capacity to express cytotoxic molecules such as *GNLY*, *GZMB*, and *NKG7* is diminished (Fig. 4g, Data S7). Gene set enrichment analysis showed that lymphocytes in myxoma were significantly associated with cell-matrix adhesion, antigen receptor-mediated, and Wnt signaling pathways (Fig. 4h, Data S8).

Next, we inferred the differentiation trajectory of CD8+ and CD4 + T cells. The results indicate that CD8 + T cells exhibit a predominant differentiation trajectory that started with T_CD8 + _C0 followed by T_CD8 + _C1, T_CD8 + _C2, and ended in T_CD8 + _C3 (Fig. 4i, Supplementary Fig. 7f). As pseudotime increases during this trajectory, *GZMB* is downregulated, *TOX* is gradually upregulated, and *GZMK* expression increases (Fig. 4j). These data indicate that CD8 + T cells transition from a precursor-like state to a cytotoxic state and finally to a dysfunctional state, and myxoma tissue is enriched with terminally evolved dysfunctional T cells. CD4 + T cells showed a trajectory that started with naive T cells, which then segregated into 3 major branches, ultimately terminating in Treg, T_CD4 + _C1, and T_CD4 + _C2, respectively (Fig. 4k, Supplementary Fig. 7g). In normal tissues, the evolution primarily consists of T_CD4 + _C1, with upregulated genes in this process being associated with cellular cytotoxicity. Conversely, in myxoma, the transition mainly involves T_CD4 + _C2, with upregulated genes associated with cytokine production during this process (Fig. 4l, Supplementary Fig. 7h).

### Macrophages in myxoma tissues exhibit a tumor-promoting phenotype

**Fig. 5:**
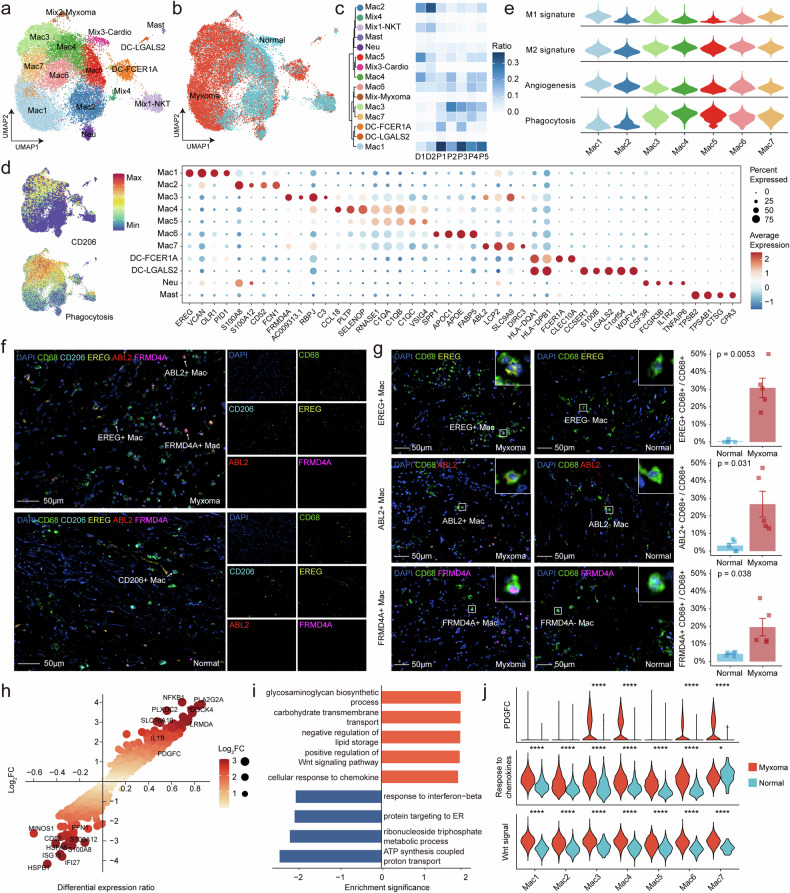
Tumor-promoting macrophage in cardiac myxomas. **a** UMAP view of myeloid cell clusters from 7 samples. **b** The UMAP plot shows the group information of all myeloid cells. **c** Dendrogram demonstrating the similarity of myeloid cell cluster centroids. The heatmap shows the proportion of each myeloid cell subset in different samples. **d** Expression levels and frequencies of selected markers across myeloid cell clusters. **e** Violin plots showing some characteristics in macrophage clusters scored by the *AddModuleScore* function. **f** The mIHC staining demonstrates the tumor-promoting macrophage in myxoma and normal tissue. Scale bars, 50 μm. **g** The mIHC staining demonstrates the *EREG*, *ABL2*, and *FRMD4A* macrophages in myxoma and normal tissue. Scale bars, 50 μm. The bar plots show the proportion of macrophages expressing *EREG*, *ABL2*, and *FRMD4A*, n = 5 in each group. The error bar indicates the standard error of the mean. **h** Scatter plot showing differential genes of myxoma and normal macrophages. Genes with corrected p values less than 0.05 in the differential analysis are displayed in the plot. The x-axis represents the difference in gene expression proportions between the two cell groups, while the y-axis represents the fold difference in gene expression means between the two cell groups. **i** Barplot shows significantly upregulated (orange) and downregulated (blue) gene sets in macrophage of myxoma as obtained from the GSEA method. **j** Violin plots showing signature scores in macrophage across myxoma and normal tissues. Cellular response to chemokine, GO:1990869; Positive regulation of Wnt signaling pathway, GO:0030177. ****P < 0.0001

We next focus on myeloid cells that play important functions in cardiac tissue. Unsupervised clustering of myeloid lineage cells from the integrated dataset revealed 7 classes of macrophages, 2 classes of dendritic cells, and smaller populations of neutrophils (Fig. 5a). Compared to the donor controls, we observed increased infiltration of Mac1, Mac3, and Mac7 in individuals with myxoma (Fig. 5b, c), and the marker genes of these macrophages were closely linked to tumor progression (Fig. 5d). Mac1 was characterized by the expression of growth factor *EREG*, which promotes tumor cell growth.^49^ Additionally, Mac1 expressed *VCAN* and *OLR1*, genes associated with tumor cell invasion and immune evasion,^50,51^ along with a higher angiogenesis score. Mac3 and Mac7 were typical M2-like macrophages, with high phagocytic activity scores (Fig. 5e). Mac3, marked by elevated expression of *FRMD4A*, *RBPJ*, and *C3*, and Mac7, characterized by increased expression of *ABL2*, likely participate in myxoma tumor proliferation, adhesion, and invasion.^52^ The mIHC staining confirmed the presence of various pro-tumor macrophage subtypes within myxoma tissue (Fig. 5f), with a significant increase in the infiltration proportion of these macrophages compared to normal tissue (Fig. 5g).

Macrophages in myxoma were associated with the upregulation of *NFKB1*, *PDGFC*, and *IL1B*, as well as the downregulation of *ATP5PB*, *HSPB1*, and *IFI27* (Fig. 5h, Data S9). The GSVA results suggested that myxoma-derived macrophage showed notable differences in multiple biological pathways associated with tumor progression, such as “cellular response to chemokine” and “positive regulation of Wnt signaling pathway” (Fig. 5i, Data S10). Although most macrophages in normal cardiac tissue exhibit an M2-like phenotype, those in myxoma display elevated expression of growth factors and activation of pro-tumor pathways (Fig. 5j).

### Macrophages promoted the proliferation of myxoma cells

**Fig. 6:**
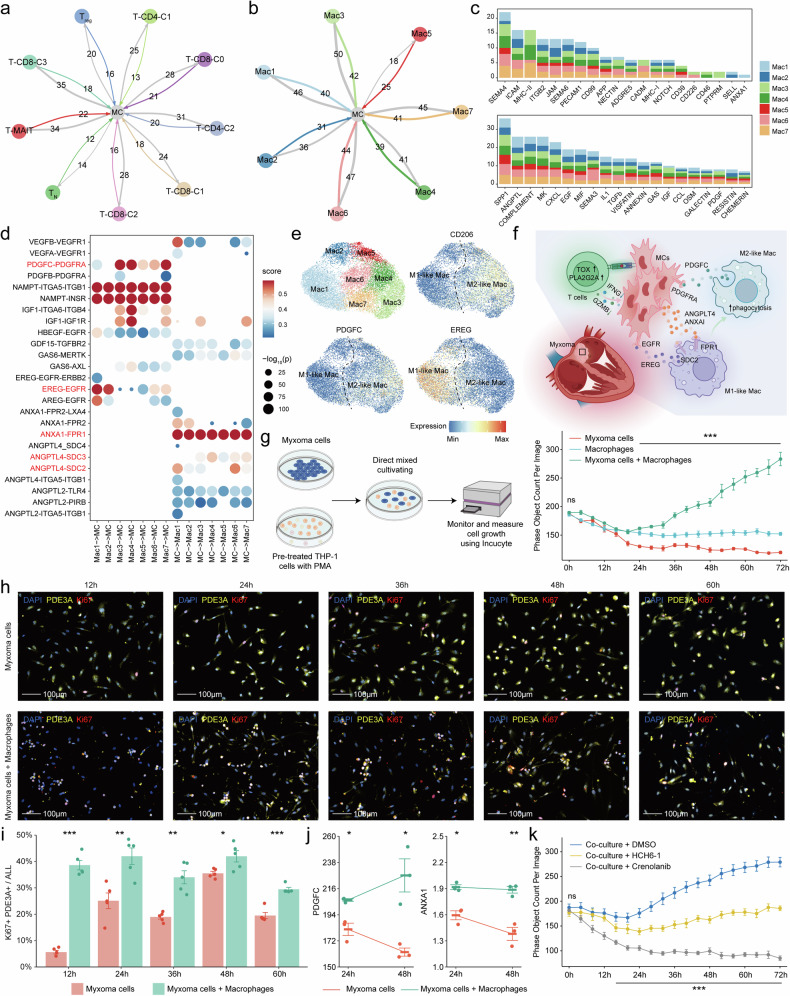
Macrophages promoted the proliferation of myxoma cells. **a** The number of significant ligand-receptor interactions between T cells and myxoma cells. **b** The number of significant ligand-receptor interactions between macrophage and myxoma cells. The direction of the arrow indicates the cell type expressing the ligand, while the direction the arrow points to represents the cell type expressing the receptor. **c** Barplot depicting the number of significant ligand-receptor interaction pairs (y-axis) between myxoma cells and macrophages (filled by different colors) in different pathways (x-axis). **d** Representative ligand-receptor pairs between myxoma cells and macrophages. The color indicates the strength of the ligand-receptor interactions, and the dot size represents the statistical significance of interactive molecular pairs. **e** The expression of genes is projected on the UMAP plot of macrophages. **f** The infographic (created with BioRender.com) summarizes predicted cell-cell interaction circuits in myxoma. **g** Incucyte proliferation assay of the co-culture system between myxoma cells and macrophages. **h** Immunofluorescence images show the proliferation of myxoma cells at different time points in the single culture and co-culture system. **i** Comparison of the number of myxoma cells in proliferative state at different time points in single culture and co-culture systems. **j** The secretion levels of some factors in a single culture and co-culture system are based on Elisa. The error bar indicates the standard error of the mean. **k** Comparison of myxoma cell proliferation status in the co-culture system after the addition of different inhibitors. HCH6-1, FPR1 antagonist. Crenolanib, PDGFR inhibitor. *P < 0.05; **P < 0.01; ***P < 0.001

We analyzed the interaction between other cells and myxoma cells based on the expression of ligand-receptor pairs in scRNA-seq data. Results showed that T cells, which are abundantly present in myxoma tissue, often exhibited stronger interactions with myxoma cells, and the communication between them primarily involves MHC-I, TGF-β, and chemokine signaling pathways (Fig. 6a, Supplementary Fig. 8a–c). Extensive interactions exist between stromal cells and myxoma cells, including signaling pathways related to cell junctions and adhesion, such as the JAM, SEMA3, and NOTCH pathways (Supplementary Fig. 8d, e).

Compared to T cells, macrophages exhibited a more active interaction with myxoma cells (Fig. 6b, Supplementary Fig. 8f). The communication between macrophages and myxoma cells involved several signaling pathways reported to promote macrophage M2-like polarization, such as ANGPT, ANNEXI, ANXA1, VISFATI, and GAS signaling pathways ^53,54^and tumor growth-promoting pathways, such as EGF and PDGF signaling pathways ^55,56^(Fig. 6c). Results showed that *EREG*, *PDGFC*, and *NAMPT*, which were secreted by macrophages, may bind to their corresponding receptors on myxoma cells to promote the proliferation and survival of myxoma cells ^49,57^(Fig. 6d), of which PDGFC was predominantly expressed in M2-like macrophages, while EREG is primarily expressed in M1-like macrophages (Fig. 6e). On the other hand, *ANXA1* and *ANGPTL4* secreted by myxoma cells may bind to their corresponding receptors on macrophages, such as *FRP1* and *SDC2*, to promote M2-like polarization of macrophages.^58,59^ These findings suggest that the interactions between myxoma cells and macrophages may sustain the growth of mucinous tumor cells (Fig. 6f).

Subsequently, we aimed to validate the pro-growth capability of macrophages on myxoma cells in vitro. Initially, we isolated CD45- cells from tumor tissues of cardiac myxoma patients, identifying them as predominantly myxoma cells (Supplementary Fig. 8g). We established a direct co-culture system of myxoma cells with macrophages, wherein the presence of macrophages significantly promoted the growth of myxoma cells (Fig. 6g, Supplementary Fig. 8h, Movies S1–3), with more cells entering a proliferative state (Fig. 6h, i). Furthermore, we concurrently assessed the secretion profile of factors in the co-culture system and found a significant elevation in the levels of *PDGFC* and *ANXA1* (Fig. 6j). Upon the addition of the *ANXA1* receptor antagonist HCH6-1 in the co-culture system, the macrophage-mediated proliferation of myxoma cells was inhibited (Fig. 6k). Additionally, the proliferation of myxoma cells in the co-culture system was significantly suppressed upon the addition of the *PDGFC* receptor inhibitor Crenolanib (Fig. 6k). These results indicate that the interaction between macrophages and mucinous tumor cells can enhance cardiac myxoma growth.

## Discussion

In this study, we utilized single-cell sequencing technology to unveil the cellular composition of cardiac myxomas, providing novel insights into the mechanisms underlying their formation. Our data reveals the characteristics of tumor cells in cardiac myxoma and delineates its immunosuppressive microenvironment characterized by T-cell dysfunction and abundant pro-tumor macrophages. The proportion of myxoma cells and the tumor-promoting macrophages in the myxoma tissues are closely linked to the severity of the disease.

In this study, we captured the comprehensive transcriptomic characteristics of myxoma cells. The abundance of marker genes in myxoma is linked to various biological processes, such as cell proliferation, differentiation, adhesion, and migration, which exhibit remarkable similarities with the hallmark characteristics of malignant tumors.^60^ Unlike the metabolic reprogramming characterized by the Warburg effect in malignant tumor cells,^61^ myxoma cells appear to favor an alternative metabolic strategy. In comparison to normal fibroblasts, myxoma cells displayed significant activation of cyclic nucleotide degradation metabolism. Specifically, the expression of the phosphodiesterase gene family, including *PDE3A*, *PDE1A*, *PDE7B*, and *PDE10A*, was markedly upregulated. The PDEs regulate cardiac function by degrading cAMP and cGMP, which are important second messengers in cells.^62^ Previous studies have shown that PDEs are dysregulated in various cardiac diseases, such as cardiac fibrosis, dilated cardiomyopathy, and ischemic heart disease.^63^ Cardiac myxomas may have distinct local pools of cyclic nucleotides to perform specialized functions. This finding provides new insights into the metabolic characteristics of myxoma cells and may have implications for the development of targeted therapies.

Similarly, our results reveal that myxoma cells possess a strong capacity for immune evasion, potentially manipulating the surrounding immune microenvironment to facilitate their growth. CD8 + T cells in the vicinity of myxoma exhibit functional impairment, characterized by high expression of *TOX* and the inflammatory molecule *GZMK*.^46,64^ Concurrently, the widespread upregulation of *PLA2G2A* in myxoma tissue may contribute to the reduction of T cell cytotoxic function and the ability to express *GZMB*.^65^ Increased infiltration of specific macrophage subtypes marked by genes that are closely associated with tumor progressions, such as *EREG*, *VCAN*, and *PDGFC* has been observed in myxoma.^49,50^ The collaborative relationship between macrophages and myxoma cells in establishing a microenvironment is conducive to tumor growth, especially the bidirectional communication between M2-like macrophages and myxoma cells. These interactions involved signaling pathways associated with macrophage M2 polarization, which is known to support tumor proliferation,^66^ as well as pathways like EGF and PDGF signaling that promote tumor cell proliferation and survival.^67^ In vitro co-culture experiments demonstrate that macrophages can promote the growth of myxoma cells. These findings underscore the necessity of an inhibitory immune microenvironment for myxoma progression.

Despite being benign tumors, cardiac myxomas often cause catastrophic clinical consequences due to embolism.^4^ The unique extracellular matrix remodeling ability of cardiac myxomas may lead to the shedding of myxoma cells, causing embolic events. We found a myxoma subpopulation, primarily located at the terminal end of one myxoma differentiation trajectory, exhibits a high expression of *PLAT*, a gene associated with cell migration and tissue remodeling. Some studies suggest that PLAT can help cancer cells breach tissue barriers by degrading the extracellular matrix, thereby promoting tumor cell invasion and metastasis.^30^ We hypothesize that myxoma cells with high *PLAT* expression may be more likely to cause embolization through extracellular matrix remodeling. This will need to be further confirmed in future studies. Furthermore, extensive multicolor immunohistochemistry experiments have indicated a significant infiltration of myxoma cells and CD206+ cells, as well as their close spatial proximity, which is correlated with the occurrence of embolisms. Co-culture experiments of myxoma cells and macrophages demonstrated that macrophages can promote the proliferation of myxoma cells. The treatment targeting *PDE3A* and M2-like macrophages could potentially serve as a conservative approach to prevent the occurrence of embolic symptoms, thereby optimizing existing treatment strategies for cardiac myxomas. Clinically, the PDE was an important target for heart failure and cardiac hypertrophy, and PDE3 inhibitors have been used to improve systolic function.^68^ Further research and investigation are warranted to determine the effectiveness of these drugs in the treatment of cardiac myxoma.

While cardiac myxomas are relatively common among cardiac tumors, their overall incidence remains low, resulting in a limited number of available samples. This limitation in sample size has been a significant drawback in our study, preventing us from conducting more detailed stratified analyses. The classification analysis of cardiac myxomas and the comparison of different types of myxomas, such as those from the left and right atria, will be important objectives for future research. Besides, none of the cases of cardiac myxoma that we collected had a family history of the disease. Therefore, it remains a challenge to generalize our research findings to the cases of cardiac myxoma with a family history, and further studies are needed in this regard. Patients with a family history of cardiac myxomas often face a higher risk of recurrence after resection,^69^ which suggests the prediction of recurrence risk is a crucial issue for these individuals. Consequently, future research should involve long-term follow-up analysis to determine the predictive value of myxoma cells and M2-like macrophage infiltration for recurrence. More importantly, the current in vivo models for cardiac myxomas are not well-developed, making it difficult to obtain direct experimental evidence of the roles of myxoma cells and M2-like macrophages in embolization. Developing in vivo models for cardiac myxomas is crucial for studying the mechanisms by which myxoma cells and M2-like macrophages promote embolization, as well as for validating therapeutic targets for cardiac myxomas.

In summary, our research reveals distinct cellular populations within cardiac myxoma tissues, highlighting the heterogeneity of myxoma cells and their collaborative influence on the immune microenvironment. These findings offer insights into the mechanism of cardiac myxoma, which discovered some potential therapeutic targets and underscored the impact of cellular interactions on cardiac myxoma progression.

## Materials and methods

### Human specimens

The study was approved by the Biomedical Ethics Review Committee of West China Hospital of Sichuan University (No. 2023746) and the Medical Ethics Committee of Xiangya Hospital of Central South University (No. 202303035). We obtained fresh cardiac myxoma samples from 5 patients for single-cell sequencing analysis. Additionally, we obtained 8 atrial septal samples from patients with mitral valve disease to serve as control and validation specimens. All patients signed an informed consent form. Meanwhile, we retrospectively collected tumor block specimens from 49 patients with cardiac myxoma at Xiangya Hospital of Central South University, which were fixed in formalin and embedded in paraffin for subsequent multi-color immunohistochemistry experiments. The requirement for inform consent was waived because of our retrospective design and no individual information was disclosed. All research processes were conducted under the regulations of the Declaration of Helsinki.

### Patients used for clinical correlation analysis

We retrospectively gathered data from 49 patients who underwent cardiac myxoma resection at Xiangya Hospital between 2019 and 2022 (Supplementary Table 2). Among these cases, 11 were incidentally diagnosed with myxoma during routine health checkups and were classified as asymptomatic patients. 8 individuals presented with evident embolic symptoms upon admission, such as cerebral and myocardial infarctions, categorized as severely symptomatic cases. The remaining 31 admitted patients primarily complained of post-exertional palpitations and dyspnea, designated as mildly symptomatic cases. We used the Wilcoxon rank-sum test to analyze differences in cell infiltration among patients with different symptoms. The logistic regression was employed to assess the association between various cell infiltrations and clinical factors.

### Preparation of single-cell suspensions

Begin by washing the tissues with PBS. Then, use sterile scissors to finely mince the tissues and place them in a tube. Add a mixture of enzymes consisting of 1 mg/ml of neutral protease, 1 mg/ml of Collagenase Type 1, 2, 4, and DNase I into the tube. Perform digestion at 37 °C with shaking for 30 min. Ensure complete digestion of any remaining undigested tissue. Collect the cell suspension by passing it through a 40 µm mesh filter after centrifugation and filtration. To the pellet obtained after centrifugation, add a red blood cell lysis solution. After some time, resuspend the cells in PBS. Take a small amount of the suspension for Trypan Blue staining and microscopic examination.^70^ Resuspend the cells in Dead Cell Removal MicroBeads, allowing them to sit at room temperature to label dead cells and cell debris. Precondition the separation column with 1× Binding Buffer Stock Solution, discard the initial 5–6 drops of liquid, and collect the subsequent liquid in a new tube. Drip the labeled cell suspension onto the separation column and wash the column 2–3 times with 1× Binding Buffer Stock Solution to ensure that all liquid passes through the column. Afterward, wash the separation column with 1× Binding Buffer Stock Solution and collect the separated cells. Resuspend the separated cells in an appropriate 0.04% BAS solution. Take a small aliquot of the cell suspension for Trypan Blue staining and microscopic examination. If there is minimal cell debris in the background, the proportion of dead cells should be around 5%, and no cell clumping should be observed. This allows for cell counting and subsequent analysis.

### Preparation of scRNA-seq libraries

We performed all steps following the 10X protocol. We used the Chromium Single Cell 3′ Library & Gel Bead Kit v3 (10X Genomics). In short, all samples and reagents were prepared and loaded into the chip. Then, we ran the Chromium Controller for droplet generation. Reverse transcription was conducted in the droplets. We recovered cDNA through demulsification and bead purification.^71^ Pre-amplified cDNA was further subjected to library preparation. Finally, libraries were sequenced on an Illumina novaseq 6000.

### Process of scRNA data

scRNA-seq data were aligned and quantified using the CellRanger toolkit v.3.1 against the GRCh38 reference genome. Then, the qualities of cells and genes were assessed by 4 standards: (1) Cells with expressed genes over 8000 or less than 400 were filtered. (2) Cells with a total UMI count over 40,000 or less than 600 were filtered. (3) Cells that have over 10% mitochondrial counts were filtered. (4) Genes expressed in fewer than 0.1% of cells within a sample were removed.

Next, we employed a combination of three methods to identify potential doublets. For each sample, we used DoubleFinder,^72^ Scrublet,^73^ and scds ^74^separately to identify doublets in the dataset, with all algorithms executed using default parameters. Assuming an increase of 0.008 in the doublet rate for every additional 1000 cells, the expected doublet rate was calculated as follows: DoubletRate = Number of cells × 0.008 × 1e-6. Based on the expected doublet rate, we assessed the classification of each cell by the three algorithms. A cell was considered a doublet and subsequently removed if it was classified as such by at least two of the algorithms. After quality control, 75,641 single cells from 5 samples and 24,467 features remained for downstream analysis. Finally, we employed the Harmony ^75^algorithm to correct batch effects between samples, allowing for the integration of single-cell data from all samples.

### Clustering and cell type identification

We utilized the R package Seurat ^76^for comprehensive single-cell analysis. To avoid unexpected noise, genes associated with mitochondria and ribosomes were excluded. The expression matrix of the integrated dataset was normalized by the *NormalizeData* function with default parameters. The *FindVariableFeatures* function was applied with default parameters to detect 2000 highly variable genes (HVGs) for the normalized matrix. The *ScaleData* function was used to scale the expression matrix with default parameters. The Top 30 PCs were calculated using the *RunPCA* function based on HVGs and the dimensionality of the dataset was reduced using the *RunUMAP* function. Nearest neighborhood graphs were built using the *FindNeighbors* function, and the community algorithm was applied for clustering using the *FindClusters* function with a resolution setting of 0.1.

We first annotated the 10 major cell types identified in our dataset based on well-known marker genes, including *CD3D*, *CD8A*, and *GZMB* for T/NK cells; *CD68* and *CD163* for macrophage; *TPSB2* for Mast cells; *PTGDS* for pDCs; *CD79A* and *IGHG1* for B and plasma cells; *COL1A1* and *DCN* for fibroblasts; *CDH5* and *VWF* for endothelial cells; *CALB* for myxoma cells; and *MKI67* and *PCNA* for proliferation clusters. We used the *FindAllMarkers* function to identify differentially expressed genes (DEGs) of each cell type as maker genes with adjusted P < 0.05 using Bonferroni correction.

### Public scRNA-seq datasets

scRNA-seq data of normal adult human hearts from published datasets were downloaded from the Human Cell Atlas (HCA) Data Coordination Platform (DCP) with accession number ERP123138.^17^ After filtering out cells with a mitochondrial gene expression proportion exceeding 10%, a total of 85,802 cells from six donors were used for subsequent integrated analysis. We combined our myxoma dataset with this normal dataset using the Harmony ^75^method to generate an integrated dataset. The integrated dataset was also applied to the Seurat ^76^pipeline for subsequent dimension reduction (top 30 PCs were selected based on 2000 HVGs), clustering (resolution = 0.3), and cell-type annotation as described above. The cell annotation information from the original dataset was also utilized to assist in annotating cells in the integrated dataset.

### Subtype analysis for major cell type

We performed a second round of clustering to further characterize subpopulations of nonimmune cells (myxoma cells, fibroblasts, and endothelial cells), T/NK cells, and macrophages. In this analysis, normal non-immune cells, T/NK cells, and myeloid cells were integrated respectively, and patients/donors with fewer than 500 corresponding cells will be excluded. In the end, a total of 7078 cells from 2 healthy donors and 11,666 cells from 4 myxoma patients were used to construct the T/NK cell atlas; 6632 cells from 2 healthy donors and 17,375 cells from 5 myxoma patients were used to construct the myeloid cell atlas; and 69,934 cells from 6 healthy donors and 41,700 cells from 5 myxoma patients were used to construct the non-immune cell atlas. Owing to the variable amount and property of cells in each major cell type, different parameters for clustering were used. For the clustering of nonimmune cells, the top 30 PCs were selected based on 2000 HVGs (resolution = 0.8). For the clustering of T/NK cells, the top 30 PCs were selected based on 1500 HVGs (resolution = 1.2). For monocytes or dendritic cells, the top 30 PCs were selected based on 1500 HVGs (resolution = 0.6).

### Differential expression analysis

We used the *FindMarkers* function based on the Wilcoxon rank-sum test to identify DEGs across two groups. P value adjustment was performed using Bonferroni correction. Genes with adjusted P < 0.05 were considered as DEGs. Gene ontology analysis of DEGs was performed using the clusterProfiler R package.^77^

### Gene signature analysis

The AUCell package ^78^was used to perform gene signature analysis based on the Hallmark gene set derived from the MSigDB database across different states of myxoma cells. We also summarized the list of characteristic genes that define T cell functions (including naive, cytotoxic, exhaustion, inflammatory, and resident signature) and macrophage functions (including M1 and M2 polarization, phagocytosis, and angiogenesis signature) in published studies.^79,80^ The scoring of cell function based on a specific gene set was performed through the *AddModuleScore* function.

### Developmental trajectory analysis of myxoma cells

We employed CytoTRACE ^35^with default parameters to assess the differentiation potential of individual states within the myxoma cells. The lineage trajectory of myxoma cells was constructed by Slingshot ^36^with a UMAP plot as the dimensionality reduction results. By integrating CytoTRACE scores, we identified the starting points of differentiation pathways (High CytoTRACE score indicates the starting point of differentiation). The TFs of different states within the myxoma cells were identified by the SCENIC Python workflow ^78^using default parameters. Subsequently, we employed the *startVsEndTest* function from the tradeseq package ^37^to identify genes and TFs associated with pseudotime changes along the differentiation pathways. The top 200 significantly ranked genes were categorized into 4 patterns of pseudo-temporal variation using hierarchical clustering.

### Cell-cell communication analysis

We collected known ligand-receptor (LR) pairs from the CellChat database.^81^ In cell type A and cell type B, we calculated the strength and significance of interactions for a given LR pair using the following approach. The expression level of ligand L with m subunits was approximated by their geometric mean, implying that the zero expression of any subunit leads to an inactive ligand. Similarly, we computed the expression level of receptor R with n subunits. Then, the interaction score of an LR pair between cell type A and cell type B was the geometric mean of average ligand expression across all cells in cluster A and the average receptor expression across all cells in cluster B. Statistical significance was then assessed by randomly shuffling the cell type labels of cluster A and cluster B respectively and repeating the above steps, which generated a null distribution of interaction score for each LR pair in each pairwise comparison between A and B clusters. After running 1000 times permutations, p values were calculated as the fraction of permuted ligand-receptor interaction scores larger than real interaction scores.

### Collection and dissociation sorting of cardiac myxoma samples

We obtained cardiac myxoma samples from West China Hospital of Sichuan University. In each tube, 1 mg/ml of neutral protease, 1 mg/ml of Collagenase Type 1, 2, 4, and Dnase I mixed enzyme solution were added. The tissue in the tube was cut into pieces with scissors until no obvious large clumps were visible. The tubes were then placed in a 37 °C incubator and shaken for approximately 30 min for digestion, resulting in a single-cell suspension. CD45 (STEMCELL, #17898) depletion kit was used to remove CD45+ cells, yielding CD45- cells. The sorted cells were cultured and expanded in DMEM containing 10% fetal bovine serum, with passaging every 4 days.

### Real-time monitoring of cardiac myxoma cell proliferation

THP-1 cells were induced with 100 ng/ml of PMA for 48 h to obtain macrophages. A 96-well plate was then used for cell seeding. The control group contained only myxoma cells or macrophages, with 4000 cells per well. The experimental group consisted of both macrophages and cardiac myxoma cells mixed at a 1:1 ratio, with a total of 4000 cells per well. In the inhibitor-related experiments, we added DMSO, the FPR1 antagonist HCH6-1 (5 μM, Selleck, Cat. No. S0547), and the PDGFR inhibitor Crenolanib (50 nM, Selleck, Cat. No. S2730) to the co-culture system. The plate was placed into an Incucyte (Sartorius) system and images were captured using a 10x objective lens, with 4 images taken per well every 4 h, continuously for 72 h, to monitor cell proliferation.

### Cell immunofluorescence

Following the same method, THP-1 cells were first induced into macrophages. Using a 24-well plate, cell climbing slices with a diameter of 14 mm were placed into the wells, and cell suspensions were added to each well, with 3 replicates per group. The control group contained only cardiac myxoma cells, with a cell count of 20,000 cells. The experimental group contained cardiac myxoma cells and macrophages mixed at a 1:1 ratio, with a cell count of 40,000 cells. Cell climbing slices were collected at 12 h, 24 h, 36 h, 48 h, and 60 h time points for immunofluorescence staining. Initially, the slices were washed three times with PBS, then fixed in 4% paraformaldehyde at room temperature for 30 min, followed by another three washes with PBS. The slices were permeabilized with a working solution containing 0.2% Triton X-100 at room temperature for 20 min, followed by three PBS washes. A mixture of primary antibodies against Ki67 (1:100, ABclonal #A20018) and PDE3A (1:300, Abnova #H00005139-M03) was added and incubated overnight at 4 °C. The next day, the plate was allowed to equilibrate to room temperature for 40 min, followed by three washes with PBS. A mixture of secondary antibodies conjugated with fluorescent dyes 488 (VICMED #VA1022) and 647 (VICMED #VA1023) was added and incubated at room temperature for 1 h, followed by three washes with PBS. DAPI (VICMED # VIC112) was added and incubated at room temperature for 5 min, followed by three rinses with distilled water. The slices were then sealed with an anti-fluorescence quenching mounting medium.

### Enzyme-linked immunosorbent assay

Collect cell culture supernatant and centrifuge at 4 °C to obtain the supernatant. Set up standard wells, zero wells, blank wells, and sample wells. Add 50 μl of different concentrations of standard solutions to the standard wells, 50 μl of sample diluent to the zero wells, leave the blank wells empty, and add 50 μl of the test sample to the sample wells. Then, add 100 μl of Horseradish Peroxidase (HRP) labeled detection antibody. Incubate in a 37 °C water bath or incubator for 60 min in the dark. Discard the liquid, pat dry on absorbent paper, and wash five times. Add 100 μl of substrate mixture to all wells. Incubate in a 37 °C incubator in the dark for 15 min. Add 50 μl of stop solution to all wells and read the absorbance (OD values) of each well at 450 nm wavelength.

### Immunochemistry and immunofluorescence staining

After surgical resection, the tissue specimens of cardiac myxoma were promptly immersed in formalin and fixed for a duration ranging from 24 to 48 h. Subsequently, standard procedures were followed, including tissue dehydration, paraffin embedding, and sectioning. Paraffin sections were baked at 65 °C for 4 h, followed by dewaxing and rehydration using xylene and graded alcohol. For antigen retrieval, either EDTA buffer (pH 9.0) was employed. The primary antibody, CD20 (Abcam 48750 T, 1:2000), was incubated at 4°C overnight. The following day, a 1-h incubation with the secondary antibody was carried out at room temperature, followed by thorough washing. DAB (3,3’-Diaminobenzidine, Cell Signaling Technology 8059) was used for visualization, followed by counterstaining with hematoxylin. Finally, the slides were baked at 37 °C for 15 min and then sealed.

The steps for antigen retrieval in the multiplex fluorescence immunohistochemistry technique were consistent with the above procedure.^82^ For antigen retrieval, either EDTA buffer (pH 9.0) or sodium citrate buffer (pH 6.0) was employed. To minimize nonspecific antibody binding, the sections were preincubated with an antibody diluent. Sequential incubation with primary and secondary antibodies was carried out following the experimental protocol. Multiplex IHC staining was performed using an Opal 7-Color Automation IHC Kit (Akoya Biosciences, Marlborough, MA), following the manufacturer’s guidelines. Following primary antibody incubation, the slides underwent secondary antibody incubation, followed by tyrosine signal amplification. Afterward, a new round of target incubation was conducted. The targets we detected included CD8 (Cell Signaling Technology 70306 S), CD3 (MAB 0740), PDE3A (Abnova H39-M03), Ki67 (Abcam 16667), CD206 (Cell Signaling Technology 24595), CD68 (Cell Signaling Technology 76437), TOX (Abcam 155768), GZMK (Proteintech 67272), GZMB (Cell Signaling Technology 46890), EREG (Bioswamp PAB52080), ABL2 (Proteintech 17693), and FRMD4A (Bioss 8235 R). After staining all target indicators, 4’,6-diamidino-2-phenylindole (DAPI) was used to label the cell nuclei.

### Analysis of cells from mIHC image

After staining, all slides were scanned using the Vectra Polaris whole slide scanner at 20X magnification. Whole slide image reading made use of QuPath software,^83^ and cell detection command (minimum area = 10 μm^2^, maximum area = 100 μm^2^, cell expansion = 1 μm) was used to identify cell locations and achieve quantitative measurement of fluorescence intensities for various channel markers. To identify positive cells for each marker, for every slide, and each marker, we first sorted the fluorescence intensity of that marker in ascending order and excluded the top and bottom 1% outliers. These plots revealed a clear point in the distribution of positive cells where the occupancy signal began increasing rapidly. To geometrically define this point, we then found the x-axis point for which a line with a slope of 1 was tangent to the curve. We defined cells above this point to be positive cells. Upon obtaining the positivity status of each marker for every cell, we can define the cell type for that cell, while the remaining cells undefined were categorized as ‘others’.

We used the G-cross function to characterize the probability distribution of *i* cells nearest to *j* cells within any given distance.^84^ The G-cross is a spatial distance distribution metric that represents the probability of finding at least one given *i* cell of any given type within an r μm radius of any *j* cell. Mathematically, the G-cross function is expressed as follows:

$${G}_{{ij}}\left(r\right)=1-{e}^{-\lambda \pi {r}^{2}}$$

The term λ is the overall density of cells of type *j* in the slide. The G-cross curves for each group were fitted using loess regression. This analysis was conducted using the spatstat R package.^85^

### Statistical analysis

All data were analyzed with R software (V4.1.3). Quantitative data are presented as mean ± SD or as the median and interquartile range, as appropriate. The Wilcoxon rank sum test was performed to compare the genes between the 2 groups. The logistic regression was employed to assess the association between cell infiltrations and clinical factors. When dealing with multiple tests, P values were adjusted by FDR correction.

### Supplementary information

Supplementary_Materials

Movies S1

Movies S2

Movies S3

Data S1

Data S2

Data S3

Data S4

Data S5

Data 6

Data S7

Data S8

Data S9

Data S10

## Data Availability

The scRNA-seq data have been deposited to the Gene Expression Omnibus database (GSE265921). The other materials related to this study were available on reasonable request. Sequencing data were processed and analyzed using publicly available R packages. The computer code used in this study was available on reasonable request.
