# Supplementary material for: Revealing the crucial roles of suppressive immune microenvironment in cardiac myxoma progression
Source: Signal Transduct Target Ther. 2024 Aug 2;9:193. doi: 10.1038/s41392-024-01912-2 (PMC11294589; doi:10.1038/s41392-024-01912-2)
Supplement: Supplementary file 1 — Supplementary_Materials [file 41392_2024_1912_MOESM1_ESM.docx]

Supplementary Materials for

Revealing the crucial roles of suppressive immune microenvironment in cardiac myxoma progression

Zedong Jiang^*^; Qianlong Kang^*^; Hong Qian^*^; Zhijie Xu^*^; Huan Tong^*^; Jiaqing Yang; Li Li; Renwei Li; Guangqi Li; Fei Chen; Nan Lin; Yunuo Zhao; Huashan Shi1^#^; Juan Huang^#^; Xuelei Ma^#^

Correspondence to: Xuelei Ma ([drmaxuelei@gmail.com](mailto:drmaxuelei@gmail.com)) or Juan Huang ([huangjuanxy@med.uestc.edu.cn](mailto:huangjuanxy@med.uestc.edu.cn)) or Huashan Shi ([shihuashan@scu.edu.cn](mailto:shihuashan@scu.edu.cn))

**This PDF file includes:**

Supplementary Fig. 1 to Supplementary Fig. 8

Supplementary Table 1 to Supplementary Table 2

Captions for Movies S1 to S3

Captions for Data S1 to S10

**Other Supplementary Materials for this manuscript include the following:**

Movies S1 to S3

Data S1 to S10


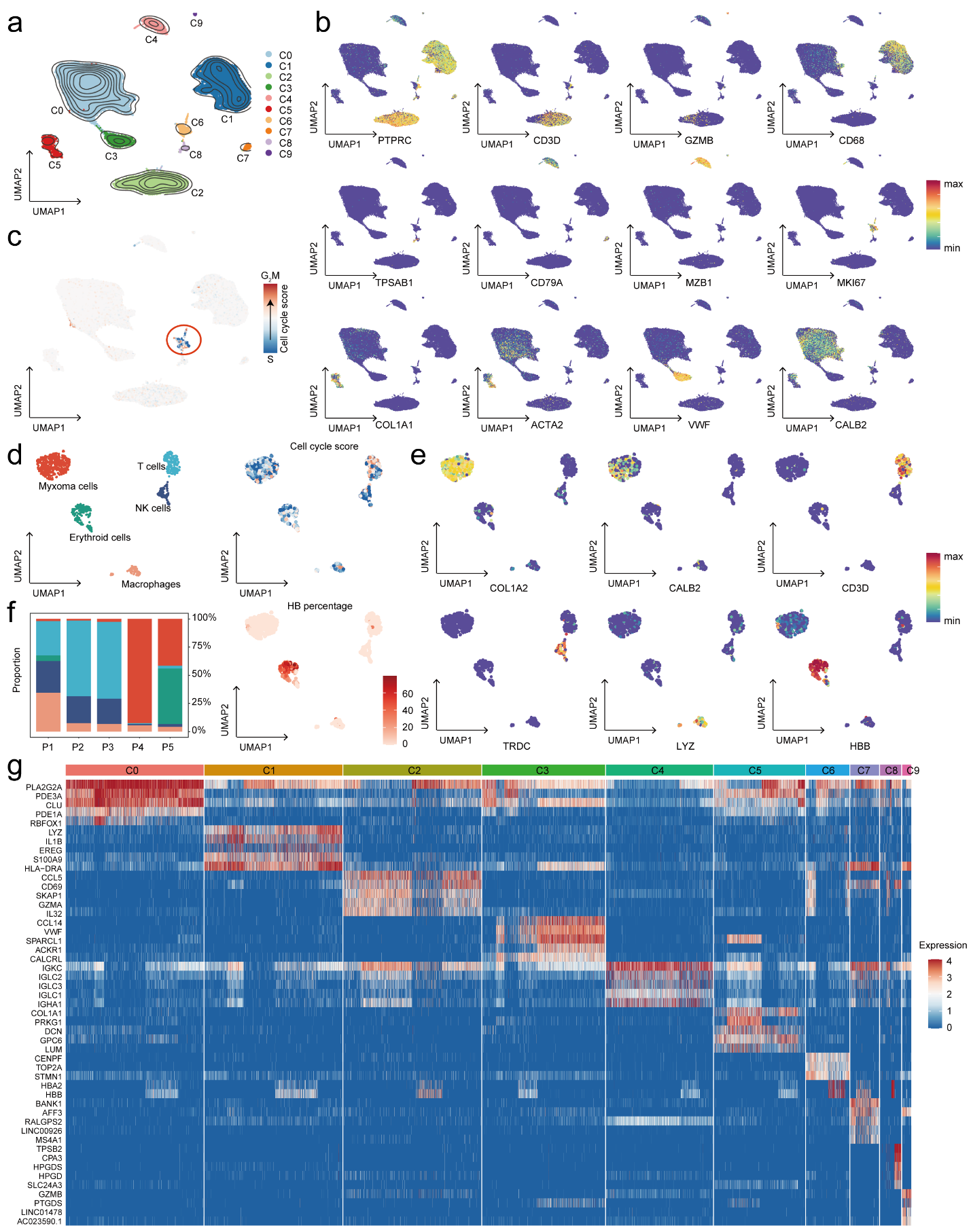


**Supplementary Fig. 1.** **Cell subtype identification for single-cell atlas of cardiac myxoma. (a)** The UMAP plot displays cell populations identified by clustering. Contour lines represent cell density. **(b)** The expression of the marker genes for the indicated cell subtypes is projected on the UMAP plot. **(c)** UMAP plot shows the distribution of cell cycle scores in cell populations. **(d)** Further cell type subdivision within the cell cycle cluster. **(e)** The expression of the marker genes for the cell subtype of the cell cycle cluster. **(f)** The bar plot shows the proportion of each cell type within the cell cycle cluster in samples. **(g)** Heatmap shows differential gene expression in various cell populations.


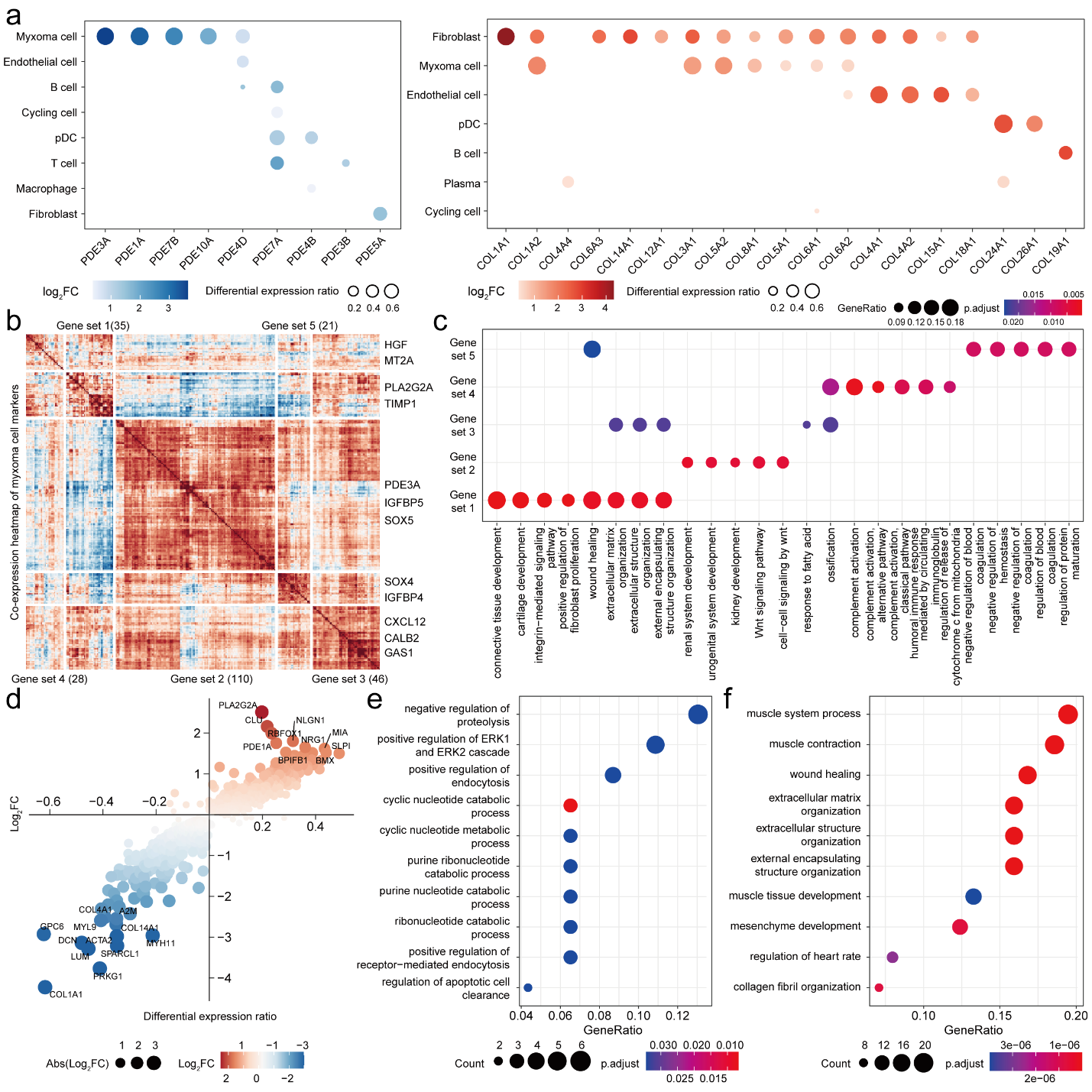


**Supplementary Fig. 2. Characteristics of the myxoma cell cluster. (a)** The bubble chart depicts the expression of the PDE gene family across different cell types. **(b)** Heatmap Illustrates the correlation among marker genes of myxoma cells. The marker genes are divided into 5 clusters based on hierarchical clustering. **(c)** Gene ontology analysis of genes of 5 clusters. **(d)** The bubble chart depicts the expression of the collagen genes across different cell types. **(e)** The scatter plot displays differential genes between myxoma cells and fibroblasts in myxoma. **(f-g)** Gene ontology analysis of upregulated **(f)** and downregulated **(g)** genes in myxoma cells compared to fibroblasts.


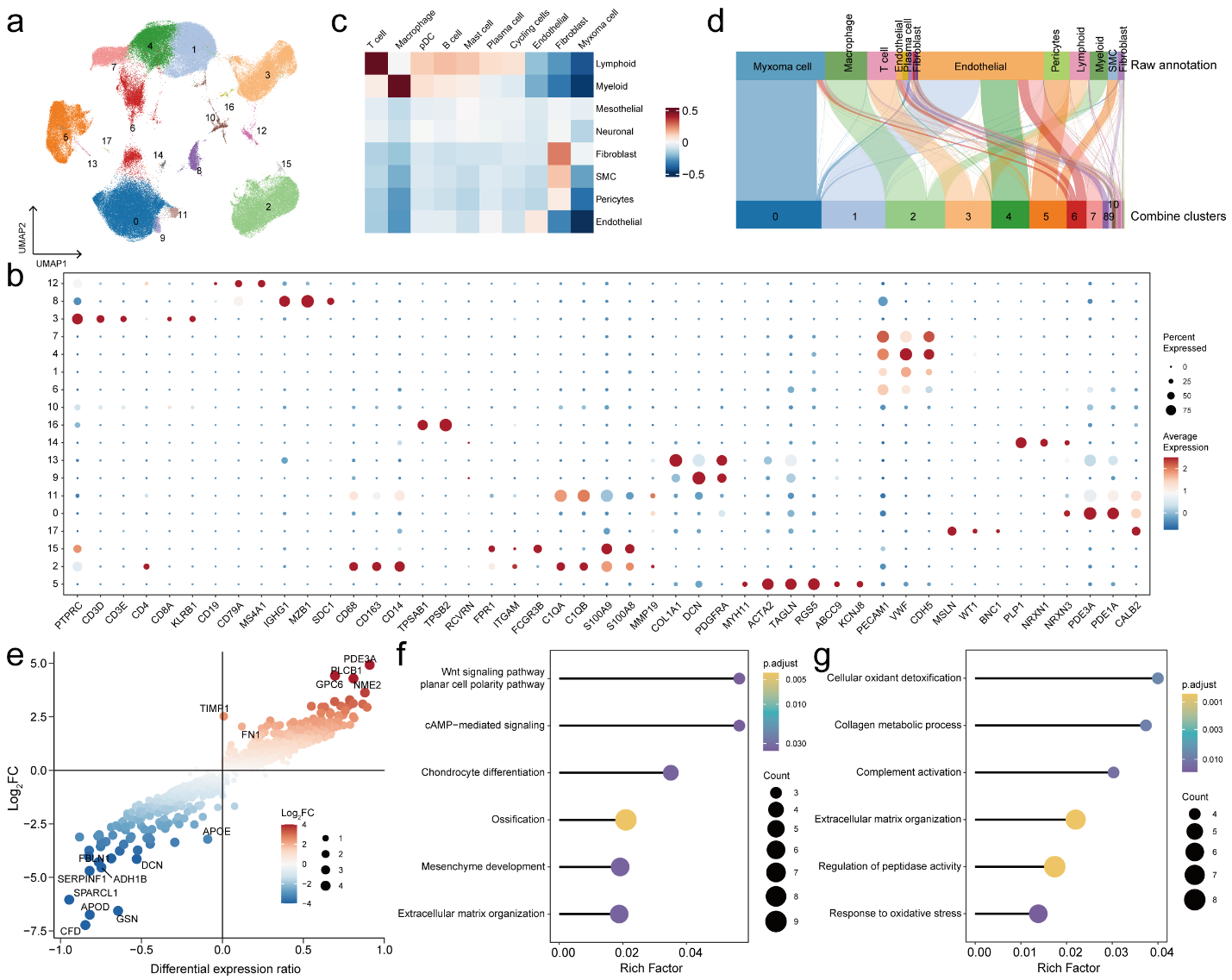


**Supplementary Fig. 3. Differences in cell composition between myxoma and normal cardiac tissues. (a)** The UMAP plot presents integrated cells from 5 myxoma samples and 2 normal samples. **(b)** The dot plot shows the expression levels of classic markers across diﬀerent clusters. **(c)** Heatmap illustrates the correlation between various cell types in myxoma and corresponding cell types in normal tissues. **(d)** The river plot shows relationships between combined cluster and raw cell annotations from separate analyses. **(e)** The scatter plot displays differential genes between fibroblasts in myxoma and normal tissue. **(f-g)** Gene ontology analysis of upregulated **(f)** and downregulated **(g)** genes of fibroblasts from myxoma compared to normal tissue.


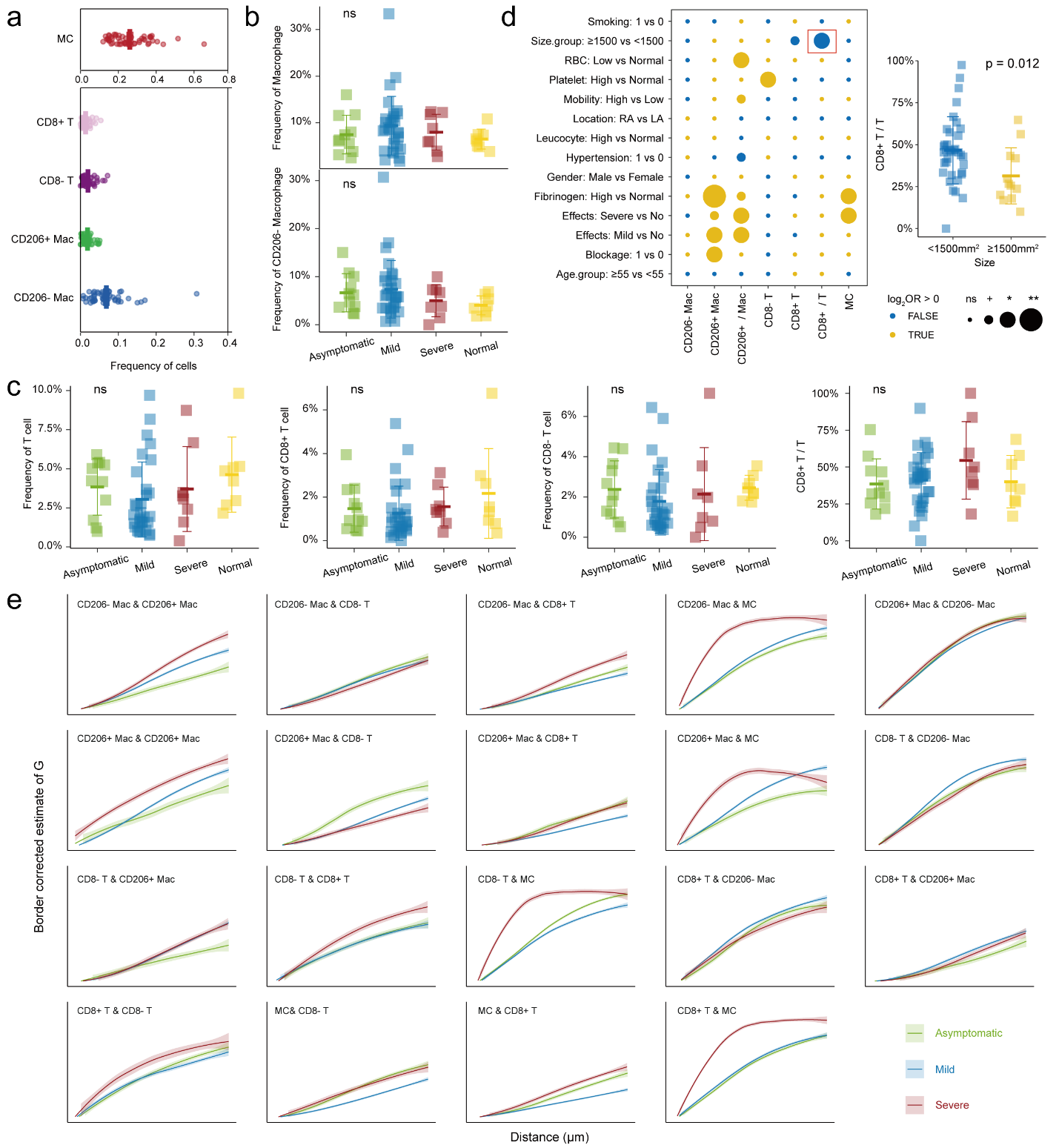


**Supplementary Fig. 4. The correlation between proportions of various cell types and clinical variables. (a)** Prevalence of 5 cell types across 49 patients as a proportion of total cells. **(b)** Prevalence of macrophage and CD206- macrophage across asymptomatic (n = 11), mild (n = 31), severe (n = 8) patients, and normal samples (n = 8). **(c)** Prevalence of other cells across asymptomatic (n = 11), mild (n = 31), severe (n = 8) patients, and normal samples (n = 8). **(d)** Bubble plot in which the circle size represents the level of significance and the circle color indicates which of the two comparisons on the y-axis has higher levels of the cell type on the x-axis (left panel). Prevalence of CD8+T / T across size subgroups (right panel). **(e)** G-cross curves of cell type *i* to cell type *j* (labeled *i* & *j*) fitted based on different severity subgroups.


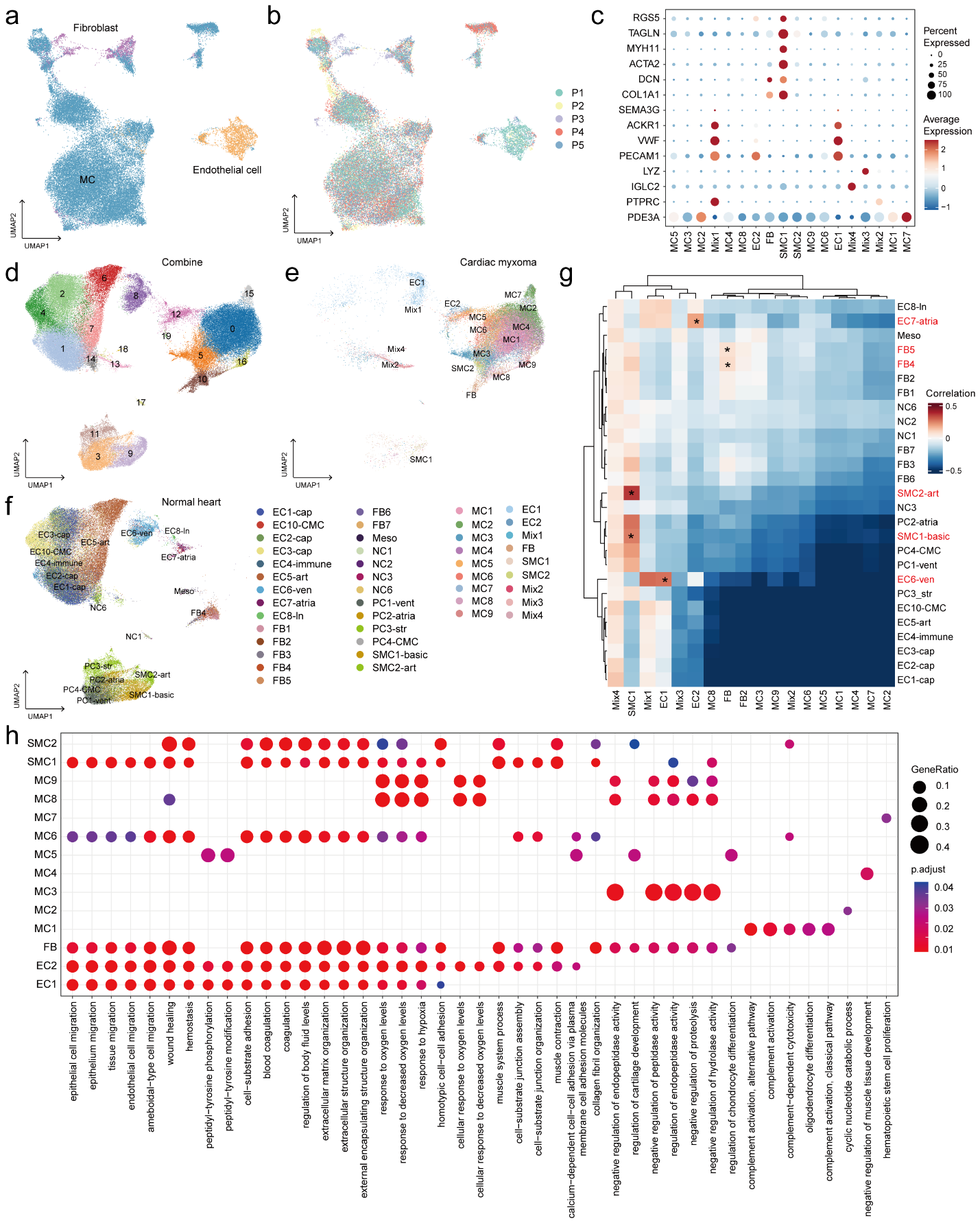


**Supplementary Fig. 5.** **Cell state analysis of nonimmune cells in cardiac myxoma. (a)** The UMAP view of nonimmune cell clusters from 5 samples. **(b)** The UMAP plot shows the sample origin of all nonimmune cells. **(c)** The dot plot shows the expression levels of classic markers across nonimmune clusters. **(d)** The UMAP plot presents integrated nonimmune cells from 5 myxoma samples and 2 normal samples. **(e-f)** The UMAP plot depicts nonimmune cell types in myxoma **(e)** and normal heart **(f)**. **(g)** Heatmap illustrates the correlation between nonimmune cell types in myxoma and corresponding cell types in normal tissues. **(h)** Gene ontology analysis of marker genes of nonimmune clusters.


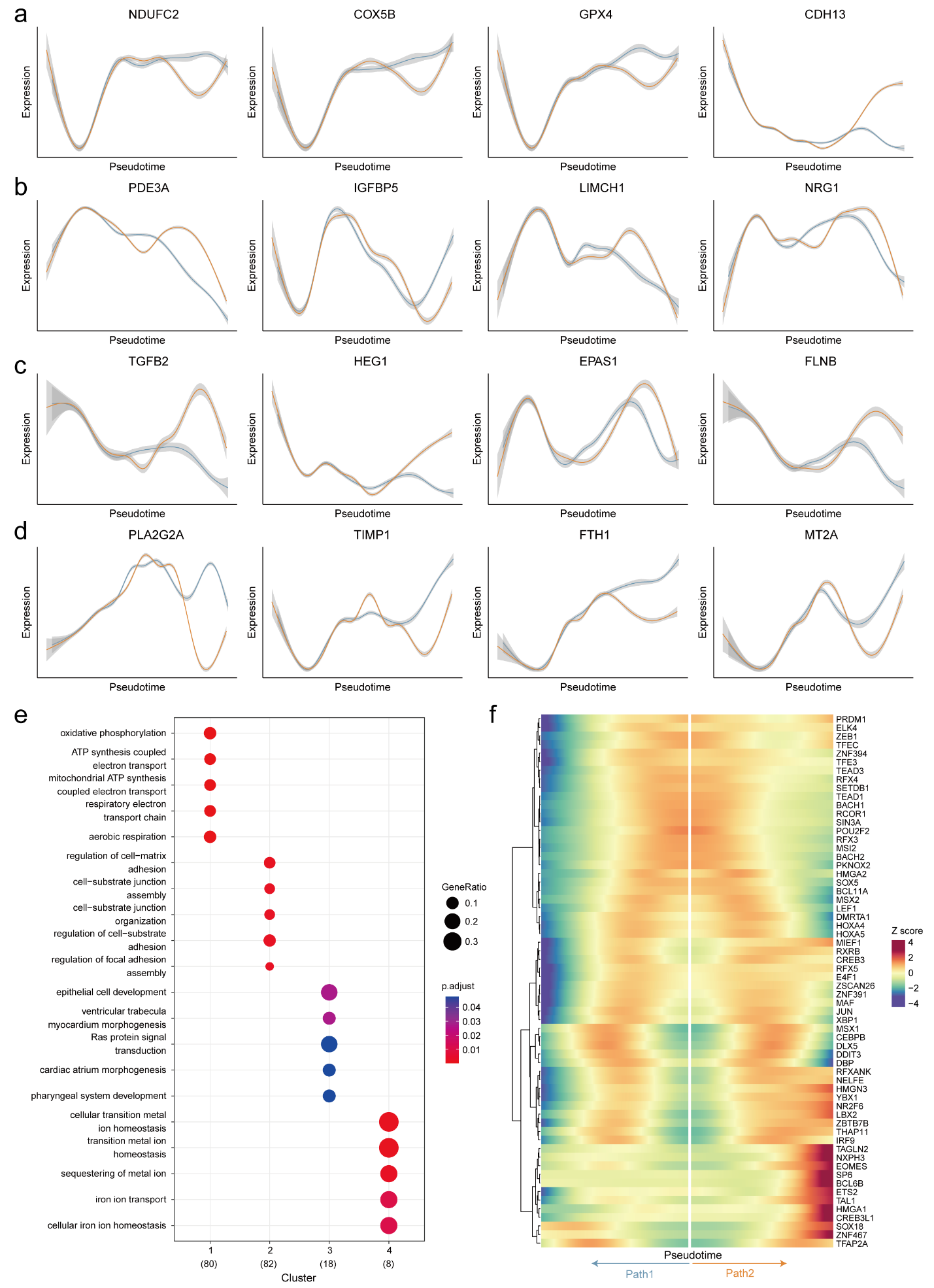


**Supplementary Fig. 6. The molecular changes associated with Evolution Pseudotime. (a-d)** Changes in genes of GS1 **(a)**, GS2 **(b)**, GS3 **(c)**, and GS4 **(d)** along pseudotime of two differentiation paths. GS1-4 are 4 gene clusters defined in Figure 2I. **(e)** Gene ontology analysis of genes of 4 gene clusters. **(f)** Heatmaps illustrate TFs linked to developmental path 1 (left) and path 2 (right).


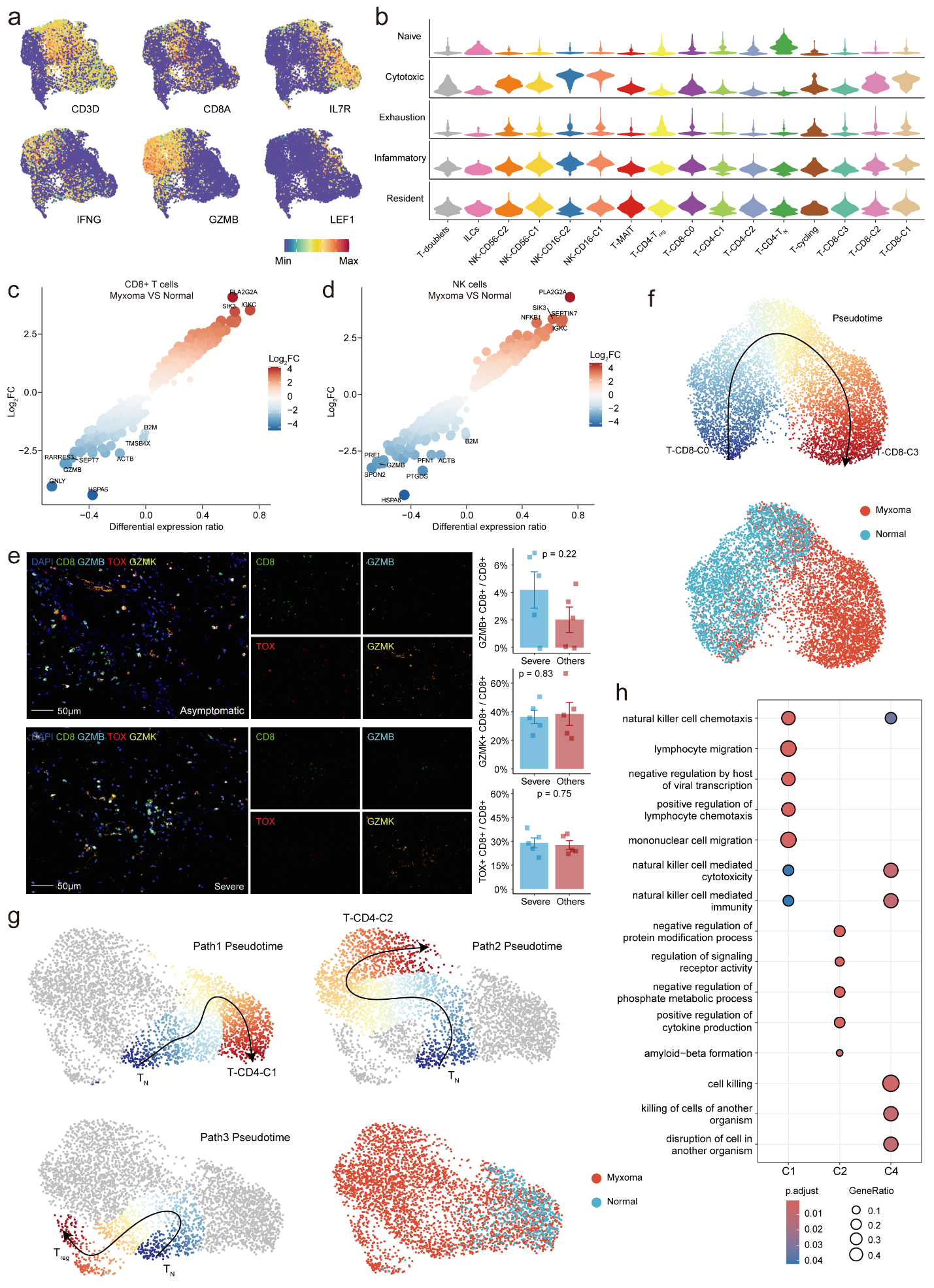


**Supplementary Fig. 7. Cell state analysis of immune cells in cardiac myxoma. (a)** The expression of the marker genes for the indicated cell subtypes is projected on the UMAP plot of lymphocytes. **(b)** Violin plots showing some characteristics in lymphocyte clusters scored by the *AddModuleScore* function. **(c-d)** Scatter plot showing diﬀerential genes of myxoma and normal tissue–derived CD8+ T cells **(c)** and NK cells **(d)**. Genes with corrected p-values less than 0.05 in the differential analysis are displayed in the plot. The x-axis represents the difference in gene expression proportions between the two cell groups, while the y-axis represents the fold difference in gene expression means between the two cell groups. **(e)** The mIHC staining demonstrates the CD8+ T cells and *TOX*, *GZMK*, and *GZMB* expression in myxoma tissue from the asymptomatic and severe patient. Scale bars, 50 μm. The bar plots show the proportion of T cells expressing *GZMB*, *GZMK*, and *TOX*, n = 5 in each group. The error bar indicates the standard error of the mean. **(f)** The pseudotime of CD8 T cells is visualized by UMAP. **(g)** The pseudotime of CD4 T cells is visualized by UMAP. **(h)** The dot plot shows the associated pathways of each gene cluster linked to CD4 T cell developmental path 1 and path 2.


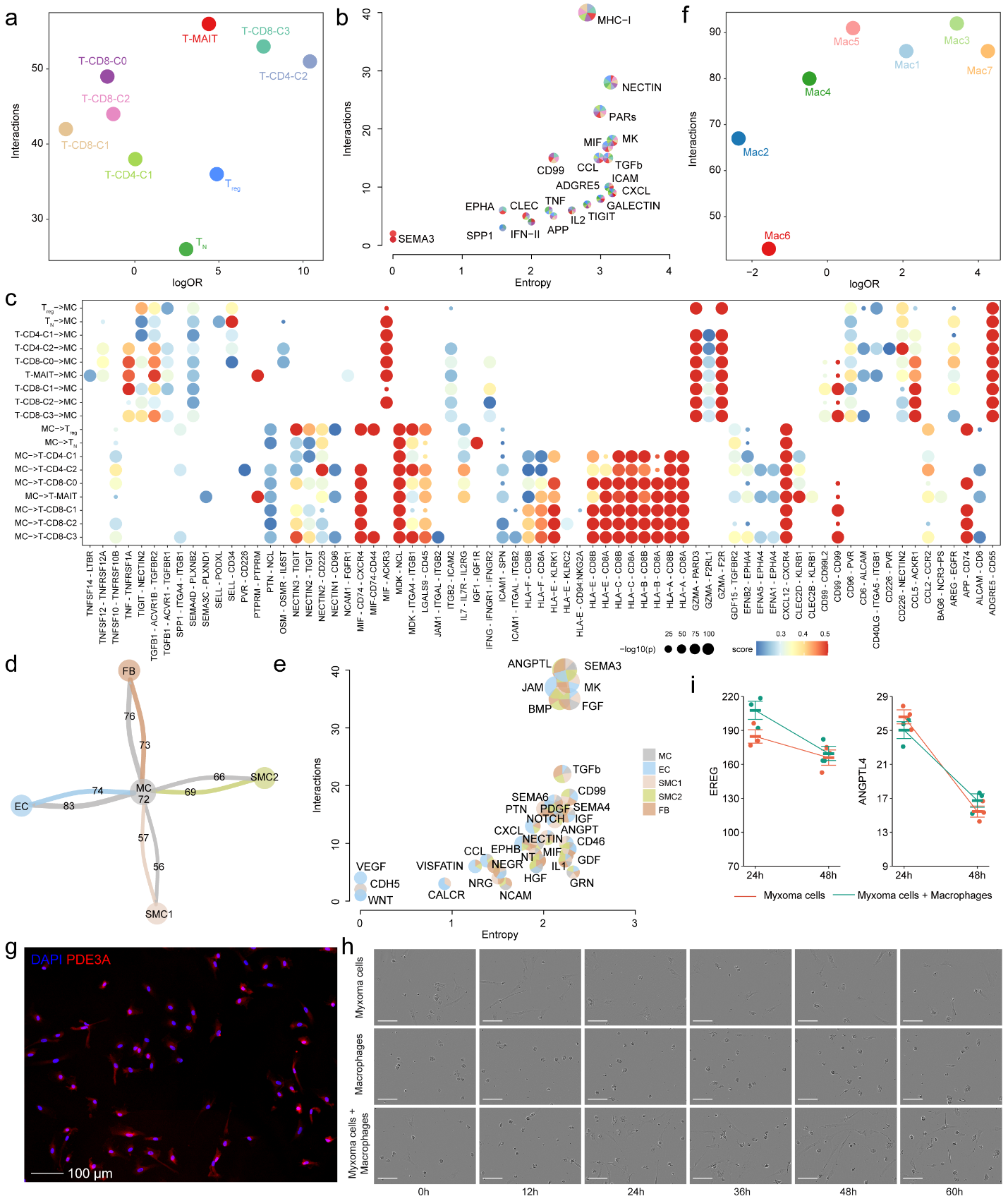


**Supplementary Fig. 8. Cell-cell interactions in cardiac myxomas. (a)** Correlation of the number of T cell interactions with myxoma cells (y-axis) and the degree of T cell enrichment in myxoma (x-axis). **(b)** The plot shows the number of significant ligand-receptor interaction pairs (y-axis) between myxoma cells and T cells (filled with different colors) in different pathways. Entropy on the x-axis represents the diversity of associated macrophage clusters in each pathway. **(c)** Representative ligand-receptor pairs between myxoma cells and T cells. The color indicates the strength of the ligand-receptor interactions, and the dot size represents the statistical significance of interactive molecular pairs. **(d)** The number of significant ligand-receptor interactions between stromal cells and myxoma cells. **(e)** The plot shows the number of significant ligand-receptor interaction pairs (y-axis) between myxoma cells and stromal cells (filled with different colors) in different pathways. **(f)** Correlation of the number of macrophage interactions with myxoma cells (y-axis) and the degree of macrophage enrichment in myxoma (x-axis). **(g)** The immunofluorescence image shows myxoma cells isolated from tumor samples obtained from patients with cardiac myxoma. **(h)** Representative images of cells during individual and co-culture systems, from the Incucyte instrument. Scale bar, 50 μm. **(i)** The secretion levels of some factors in a single culture and co-culture system are based on Elisa. The error bar indicates the standard error of the mean.

**Supplementary Table 1. Myxoma patients used for scRNA-seq**

| **Id** | **Gender** | **Age** | **Location** | **Length (mm)** | **Width (mm)** |
| --- | --- | --- | --- | --- | --- |
| P1 | Male | 79 | LA | NA | NA |
| P2 | Male | 50 | RA | 59 | 29 |
| P3 | Female | 51 | LA | 41 | 20 |
| P4 | Male | 53 | LA | 64 | 26 |
| P5 | Female | 51 | RA | 93 | 47 |

LA: left atrium; RA: right atrium.

**Supplementary Table 2. Patient information for mIHC samples.**

| **Id** | **Effect** | **Gender** | **Age** | **Hypertension** | **Smoke** | **Location** | **Length (mm)** | **Width (mm)** | **Mobility** | **Blockage** |
| --- | --- | --- | --- | --- | --- | --- | --- | --- | --- | --- |
| M1 | Severe | Female | 49 | 0 | 0 | LA | 30 | 17 | High | 0 |
| M2 | No | Female | 35 | 0 | 0 | LA | 28 | 16 | Low | 0 |
| M3 | Mild | Male | 49 | 1 | 1 | LA | 63 | 22 | High | 0 |
| M4 | Severe | Female | 54 | 0 | 0 | LA | 38 | 21 | High | 0 |
| M5 | Mild | Male | 64 | 0 | 1 | LA | 19 | 18 | High | 0 |
| M6 | Mild | Female | 64 | 0 | 0 | LA | 49 | 32 | High | 1 |
| M7 | No | Male | 70 | 0 | 0 | LA | 47 | 22 | High | 1 |
| M8 | Severe | Female | 50 | 0 | 0 | LA | 50 | 27 | High | 1 |
| M9 | Mild | Female | 44 | 0 | 0 | LA | 14 | 18 | Low | 0 |
| M10 | Mild | Female | 50 | 0 | 0 | LA | 65 | 32 | High | 1 |
| M11 | No | Male | 72 | 0 | 1 | LA | 34 | 23 | Low | 0 |
| M12 | Mild | Female | 53 | 0 | 0 | LA | 53 | 30 | High | 1 |
| M13 | No | Female | 63 | 1 | 0 | LA | 14 | 12 | Low | 0 |
| M14 | Mild | Female | 68 | 0 | 0 | LA | 23 | 23 | Low | 0 |
| M15 | Mild | Male | 67 | 0 | 1 | LA | 43 | 17 | High | 0 |
| M16 | Mild | Female | 56 | 0 | 0 | LA | 58 | 29 | High | 1 |
| M17 | Mild | Female | 52 | 0 | 0 | LA | 16 | 29 | Low | 0 |
| M18 | Severe | Male | 69 | 0 | 1 | LA | 31 | 20 | Low | 0 |
| M19 | Mild | Female | 63 | 0 | 0 | LA | 27 | 14 | High | 0 |
| M20 | Mild | Female | 47 | 0 | 0 | LA | 47 | 31 | High | 1 |
| M21 | Mild | Female | 57 | 0 | 0 | LA | 34 | 35 | High | 0 |
| M22 | Mild | Female | 51 | 0 | 0 | LA | 28 | 34 | High | 1 |
| M23 | Mild | Female | 51 | 1 | 0 | LA | 22 | 22 | Low | 0 |
| M24 | Mild | Female | 66 | 0 | 0 | RA | 51 | 36 | High | 1 |
| M25 | Severe | Female | 56 | 0 | 0 | LA | 36 | 26 | High | 1 |
| M26 | Mild | Male | 56 | 0 | 1 | LA | 33 | 46 | High | 1 |
| M27 | Mild | Male | 43 | 0 | 0 | LA | NA | NA | High | 1 |
| M28 | No | Female | 21 | 0 | 0 | RA | 58 | 29 | High | 1 |
| M29 | Severe | Female | 66 | 1 | 0 | LA | 22 | 48 | High | 1 |
| M30 | No | Female | 58 | 1 | 0 | LA | 11 | 9 | Low | 0 |
| M31 | No | Female | 64 | 1 | 0 | LA | 58 | 35 | High | 0 |
| M32 | Mild | Female | 52 | 0 | 0 | LA | 43 | 26 | High | 1 |
| M33 | Mild | Female | 46 | 0 | 0 | RA | 25 | 26 | Low | 0 |
| M34 | Mild | Female | 61 | 1 | 0 | LV | 17 | 9.1 | High | 0 |
| M35 | Mild | Female | 51 | 0 | 0 | LA | NA | NA | Low | 0 |
| M36 | Mild | Male | 53 | 0 | 1 | RA | 87 | 56 | High | 1 |
| M37 | No | Male | 78 | 1 | 1 | LA | 61 | 26 | High | 1 |
| M38 | Mild | Female | 63 | 0 | 0 | LA | 42 | 33 | High | 1 |
| M39 | Mild | Male | 57 | 0 | 0 | LA | 21 | 23 | Low | 0 |
| M40 | Mild | Female | 50 | 0 | 0 | LA | 44 | 29 | High | 1 |
| M41 | No | Female | 56 | 0 | 0 | LA | 32 | 21 | High | 0 |
| M42 | Mild | Female | 53 | 0 | 0 | LA | 25 | 19 | Low | 0 |
| M43 | Mild | Male | 69 | 0 | 0 | RA | 75 | 53 | High | 1 |
| M44 | Severe | Male | 53 | 0 | 1 | LA | 50 | 17 | High | 0 |
| M45 | No | Female | 47 | 0 | 0 | LA | NA | NA | NA | NA |
| M46 | Mild | Female | 48 | 0 | 0 | LA | 38 | 30 | High | 0 |
| M47 | Severe | Female | 57 | 1 | 0 | LA | 33 | 27 | High | 0 |
| M48 | Mild | Female | 49 | 0 | 0 | LA | 22 | 17 | Low | 0 |
| M49 | No | Female | 48 | 0 | 0 | LA | 13 | 11 | Low | 0 |

LA: left atrium; RA: right atrium; LV: left ventricular.

Movies S1. (separate file)

The real-time culture video of isolated myxoma cells was recorded using the Incucyte instrument.

Movies S2. (separate file)

The real-time culture video of macrophages was recorded using the Incucyte instrument.

Movies S3. (separate file)

The real-time co-culture video of myxoma cells with macrophages, captured using the Incucyte instrument.

Data S1. (separate file)

Marker genes of major clusters.

Data S2. (separate file)

Functional enrichment analysis of MC marker genes.

Data S3. (separate file)

Differential genes in cardiac myxoma tissue fibroblasts compared to normal tissue fibroblasts.

Data S4. (separate file)

Cells information of mIHC samples.

Data S5. (separate file)

Marker genes of non-immune clusters.

Data S6. (separate file)

Genes with variation along pseudotime.

Data S7. (separate file)

Differential genes in lymphocytes from myxoma versus normal heart.

Data S8. (separate file)

Functional enrichment analysis of differential genes in lymphocytes from myxoma versus normal heart.

Data S9. (separate file)

Differential genes in macrophage from myxoma versus normal heart.

Data S10. (separate file)

Functional enrichment analysis of differential genes in macrophage from myxoma versus normal heart.
